# Transecting and contrasting the feeding designs of the astigmatan community from bird nests

**DOI:** 10.1007/s10493-025-01014-w

**Published:** 2025-04-15

**Authors:** Clive E. Bowman

**Affiliations:** Mathematical Institute, University of Oxford, Oxford, OX2 6GG UK

**Keywords:** Acariformes, Chelicerae, Composite tools, Ecomorphology, Morphotypes, Nidicolous habitat, Piercing, Riemannian space, Weaponry

## Abstract

The chelal moveable digit patterns of seventeen free-living astigmatan mites commonly found in bird nests is decomposed (for the first time) into functional groups using standardised profiles. Contrasts along the mastication surface are used to detect trophic features so as to explain the coexistence of different species in that community. Variation in profiles in general track geometric similarity changes in chelicerae and chelae, except in the moveable digit design transition between *Thyreophagus entomophagus* TH3 and *Lepidoglyphus destructor* G6. Full-kerf (*Aleuroglyphus ovatus* AL2 and *Chortoglyphus arcuatus* CH1) and particularly thin-kerf (*Acarus farris* A17) species are found. Both the moveable ‘digit tip angle’ and the angular bluntness of the anterior region (on which the tip sits, denoted the ‘distal digit angle’), mirror digit robustification.Ventral surface intrinsic curvature of the moveable digit appears common across species. *Acarus gracilis* A4, *Glycyphagus domesticus* G5 and *Lepidoglyphus destructor* G6 have more than expected strengthened digit tips compared to other taxa. Rates of this strengthening with chelal occlusive force varies across species. With respect to the whole moveable digit profile a design transition from glycyphagids through acarids to pyroglyphids is found, along with an evolutionary path amongst pest species (*Rhizoglyphus robini* R1, through *Tyrophagus longior* T40, to *Tyrophagus putrescentiae* T13). *Acarus gracilis* A4 appears unique. In particular

- *Tyrophagus palmarum* T17 & T32 and *Tyrophagus similis* T21 & T44 are indistinguishable from replicates of each other and typify a basal form
- *Tyrophagus longior* T40, *Tyrophagus putrescentiae* T13, *Acarus immobilis* A1, *Tyrolichus casei* T62 and *Acarus farris* A17 are only mildly different from the observed scale of sampling variation of the basal overall profile form in this study
- Two design groups of ever increasing post-horizontal ramus investment are clear, with the basal rami of *Chortoglyphus arcuatus* CH1, *Thyreophagus entomophagus* TH3, *Rhizoglyphus robini* R1, *Glycometrus hugheseae* G3 and *Dermatophagoides pteronyssinus* D3 being taller and sometimes more rounded than those of the distinct group *Acarus gracilis* A4, *Suidasia pontifica* S5, *Glycyphagus domesticus* G5, *Lepidoglyphus destructor* G6 and *Aleuroglyphus ovatus* AL2.

The bulk of the bird nest astigmatan species have a common profile pattern of apparent asperities on their mastication surface. Although, two species, *Rhizoglyphus robini* R1 and *Chortoglyphus arcuatus* CH1, have somewhat exaggerated features on this common ‘Bauplan’ (perhaps scaled for greater adductive force). Certain species: *Acarus immobilis* A1, *Dermatophagoides pteronyssinus* D3, *Glycometrus hugheseae* G3, *Glycyphagus domesticus* G5, *Lepidoglyphus destructor* G6 and *Tyrophagus putrescentiae* T13, have an individualised distinctly featured mastication surface. These species must each feed differently or on different material in bird nests. Basal ramus and chelal leverage differences are discussed. More work on the ascending ramus and specific dentition in future work is needed to explain certain remaining mite coexistences in this habitat.

## Introduction

The fundamental engine of discovery is the recognition and exploration of patterns. These may be patterns in morphology or patterns in ecology. As Swartz et al. (2003) cogently points out, ”Interconnections between morphological design and function are central to biology; they underlie natural patterns in species distribution, phylogenetic diversification and morphological specialization. At its core, ecomorphology explores the causal relationships between organismal design and behavioural performance and investigates how these relationships influence an organism’s ability to exploit its environment.”. This is the crux of the acarine ecomorphological results presented below.

**Fig. 1:**
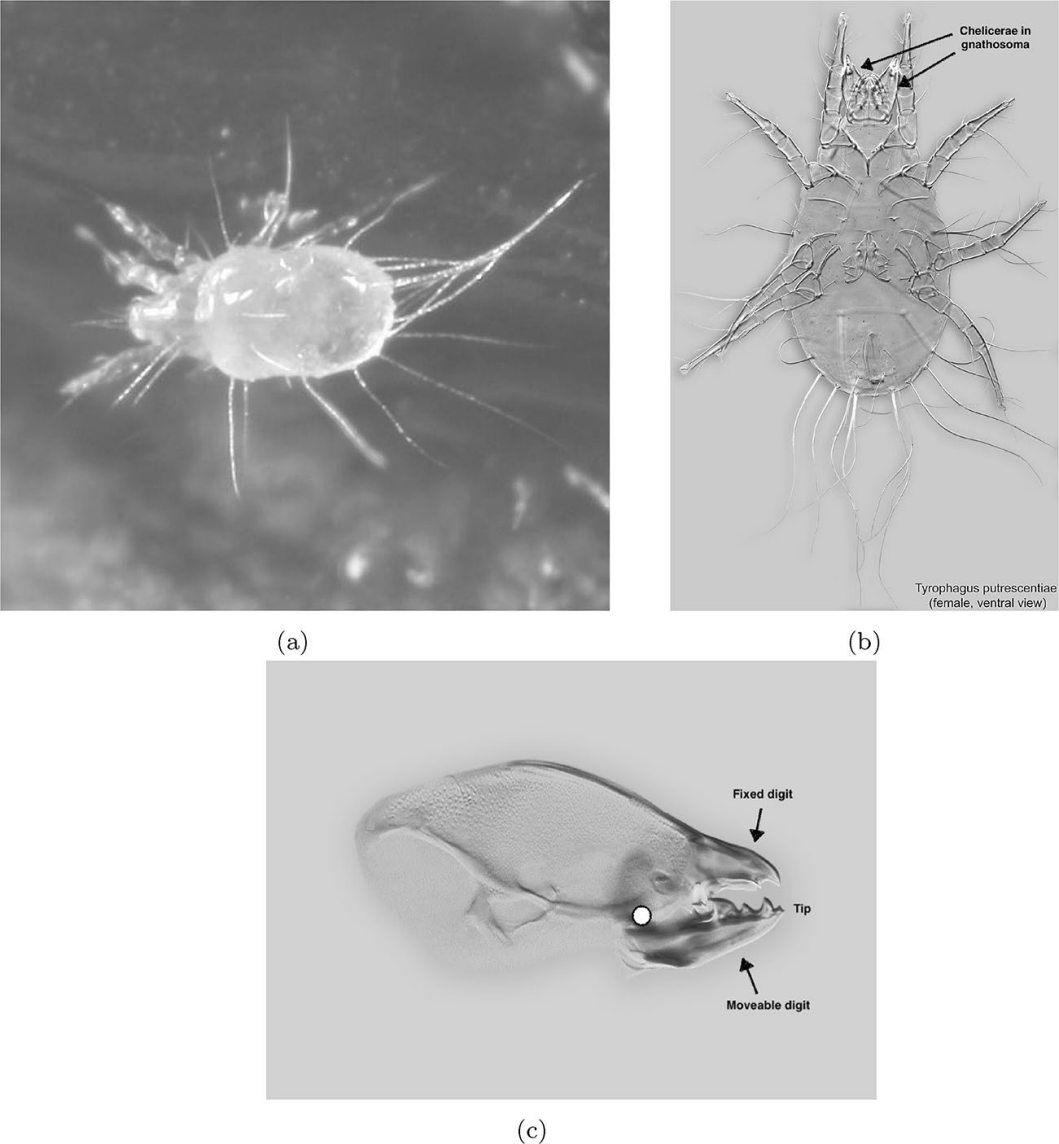
Example acarid astigmatan (illustration reproduced from Bowman (2024b) with permission, Creative Commons Attribution 4.0 International License http://creativecommons.org/licenses/by/4.0/). Note these used to be called ’astigmatids’. **a** Dorsal view of *Tyrophagus* sp. in a tortricid moth (probably *Episimus argutanus*) witch-hazel (*Hamamelis virginiana*) leaf-roll, Pelham, Hampshire County, Massachusetts, USA, August 3, 2013 ©2013 Charley Eiseman with permission. Gnathosoma to the left (partly hidden in klinorhynchid pose). **b** *Tyrophagus putrescentiae* female. Note birefringent cheliceral chelae anteriorly. Amended from a photo by Pavel Klimov, Bee Mite ID (idtools.org/id/mites/beemites) with permission. **c** Enlarged lateral view of a chelicera of *Chaetodactylus krombeini*. Note dentate chela to right end of cheliceral shaft. Tendons and musculature inside the cheliceral base actuate the (lower) moveable digit against the (upper) fixed digit (comprising the chela) around the articulating condyle (indicated by white circle). The gleaming actinochitinous nature of the digits points to their evolutionary origin from setae/ambulacra (Grandjean 1947). From colour photograph ex Pavel Klimov with permission

Morphologically, free-living astigmatan mites (Fig. 1) show many of the axes of divergence (e.g., ‘body’, ‘head’, ‘jaw’ and ‘teeth’ shape variation) that other well known evolutionary model species like the cichlid fishes do (Santos et al. 2023). Analogous structures (for the first three of these) may have different acarine names i.e., ‘idiosoma’, ’proterosoma’, ‘chelicera’, but the principles are the same. Even mite chelal digits have peaked asperities often called ‘teeth’. Indeed these mites offer some advantages to a researcher, like their ubiquity world-wide and their presence in a variety of ecosystems. Some of these feature mammalian and avian nest dwelling. As de Lillo et al. (2001) says: ”As a rule, structural and functional adaptations of the gnathosoma have evolved on the basis of mite feeding mechanisms and they can help in understanding mite trophic relationships”. Many astigmatans are nidicolous where debris of dead material may arise. Indeed, a possible necrophagous origin for some astigmatan species has been pointed to (Bowman 2021).

Nests are designed by birds for a diversity of reasons Mainwaring et al. (2014). Woodroffe (1953) classifies nests as wet or dry depending upon their exposure or protection from rain or drainage water. For sure, Woodroffe’s work shows how humidity conditions are important in determining the insect fauna found in them. Nest location rather than the exact bird species *per se* occupying it is the key. Hughes (1976) lists a variety of non-histiostomatid astigmatan (formerly astigmatid) mites common in bird nests (Table 1). Bird nests are a distinct habitat, different in its scavenging acarine composition than say cadavers (which are dominated by *Acarus siro*, *Sancassania berlesei*, *Lardoglyphus zacheri* and *Tyrophagus putrescentiae*, Braig and Perotti 2009), only the last species of which is recorded as being nidicolous.

Biotic communities are structured by drivers at various scales and levels of complexity. The research herein into acarine community ecology concentrates on mid- and fine-scale trophic morphology extending the methods of Bowman (2023a, 2024b). One might criticise the use of Hughes (1976), a treatise about pests of stored food and houses, as a source of avian nidicolous species to use in this study. However, for at least uropodine mesostigmatids, forest floor wood warbler bird nests do not contain any specialized nidicoles (Napierała et al. 2021). Indeed, *Acarus immobilis*, *Glycyphagus domesticus*, *Tyrophagus longior* and *Tyrophagus palmarum* are confirmed as common in bird nests by Solarz et al. (2004). Other mites were also found including *Rhizoglyphus robini* and *Tyrolichus casei*. Specimens of *Tyrophagus longior*, *Tyrophagus palmarum* and *Tyrophagus similis* have been found (along with *Thyreophagus* sp.) in sea-bird nests (Fain and Galloway 1993). *Suidasia pontifica* (=*Suidasia medanensis*) was found synanthropically along with *Tyrophagus putrescentiae*, *Glycyphagus domesticus*, *Lepidoglyphus destructor*, *Glycometrus hugheseae* = *Austroglycyphagus geniculatus* and *Dermatophagoides pteronyssinus* in bird nests in India (Chaudhury et al. 2012). The latter authors also report *Glycyphagus ornatus* (commonly found in beech soils by Luxton 1995). In South Brazil, Silva et al. (2018) found *Tyrophagus putrescentiae* and *Dermatophagoides pteronyssinus* in bird nests there. Free-living astigmatans are even found in edible bird nests formed from the regurgitated saliva of swifts (Kew et al. 2015). Indeed bird nests are the presumed origin of many domestic pest species (Woodroffe 1953; Woodroffe and Southgate 2009) rather than soil or plants.

**Fig. 2:**
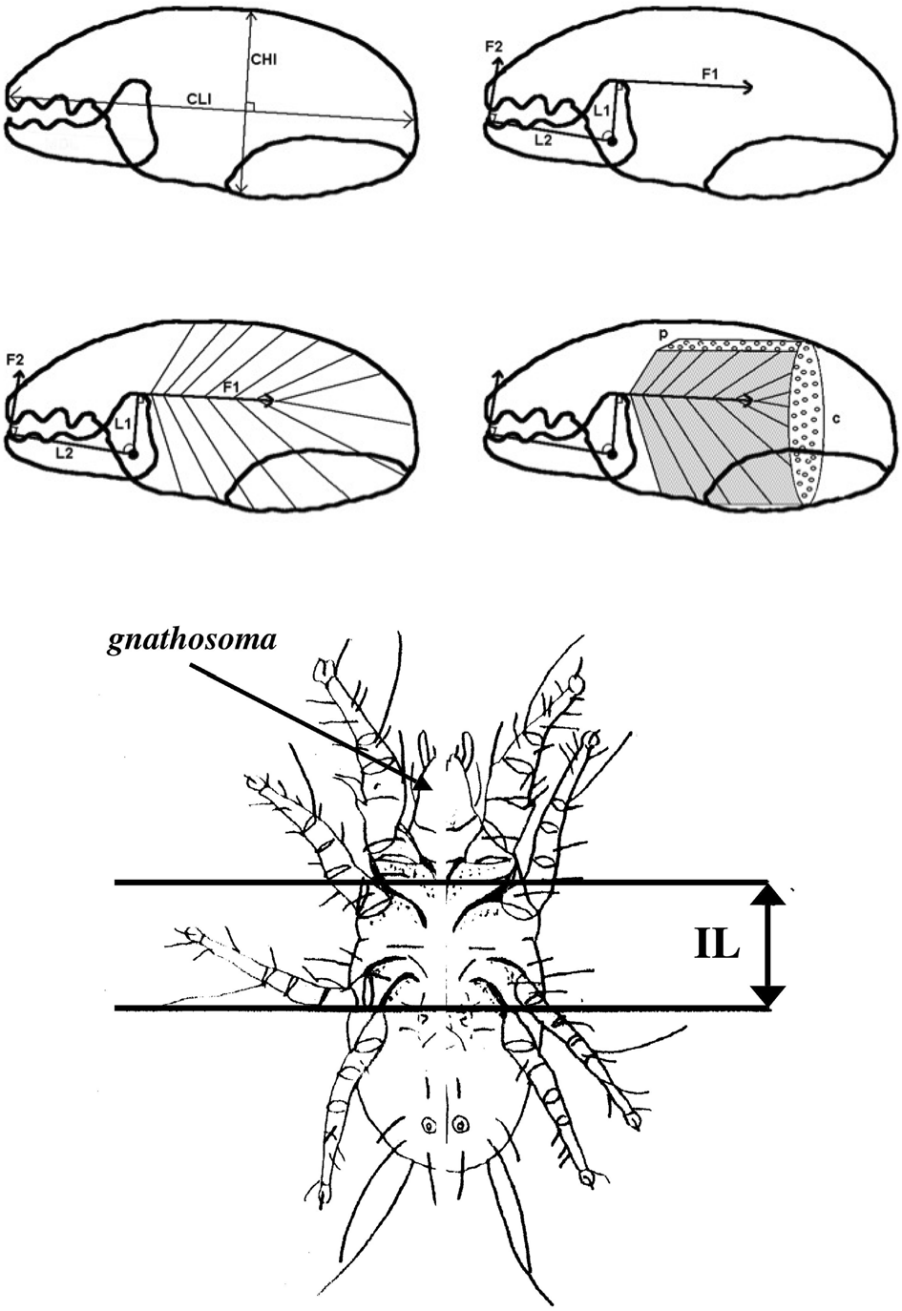
Mite mechanical model of static forces based upon rigid levers and mite size definition used in this review (reproduced from Bowman 2021 with permission, Creative Commons Attribution 4.0 International License http://creativecommons.org/licenses/by/4.0/). Upper. Stylised chelicera after Knülle (1959), showing measurement of: *Top row Left*: Cheliceral parameters (length *CLI* $\equiv$ reach, height *CHI*). *Top row Right*: Chelal parameters (fixed digit upper input lever arm L1, moveable lever output arm L2 $\approx$ gape, chelal crunch force *F*2). *F*1 is the estimated force on the adductive tendon due to cheliceral musculature. The rigid moveable digit rotates on chelal closing around a condyle (small circle) inside the chelicera actuated by musculature attached to the tendon. *Middle row*: Schema showing two assumptions of closing muscle topology. *Left*: Cheliceral base full of fibres. *Right*: p = pennate force $F1P \propto CHI*CLI$, c = circular force $F1C \propto CHI^2$ (Perdomo et al. 2012), used in calculating the adductor static force *F*1 as dependent upon a nominal cheliceral muscle cross-sectional area. Final crunch forces F2P and F2C are obtained by pre-multiplying with the velocity ratio $\frac{L1}{L2}$. Then in Bowman (2021) $F2AV=\frac{F2P+F2C}{2}$. Lower. Measurement of index of idiosomal length (*IL*) - amended after Griffiths et al. (1990). This intercoxal distance is indicative of overall idiosomal length ($\approx$ size of the mite) and is not prone to distortion on slide mounting

Feeding structures in Chelicerates comprise amongst other parts, cheliceral chelae (de Lillo et al. 2001). These jaw-like structures (comprising a moveable digit occluding against a fixed digit) are important for understanding an animal’s ethology because they operate at the direct crucial point of contact between the arthropod and its environment. All animals must feed. Bowman (2020, 2021) introduces the chelal velocity ratio $VR=\frac{L1U}{L2M}$ (which is $\equiv$ ‘ideal’ mechanical advantage, Smith and Savage 1959), where *L*1*U* is the input moment lever arm for digit occlusion and *L*2*M* is the output lever moment arm (in effectively a class 1 lever system), in order to understand moveable digit adaptations in free-living mites (Fig. 2).

**Fig. 3:**
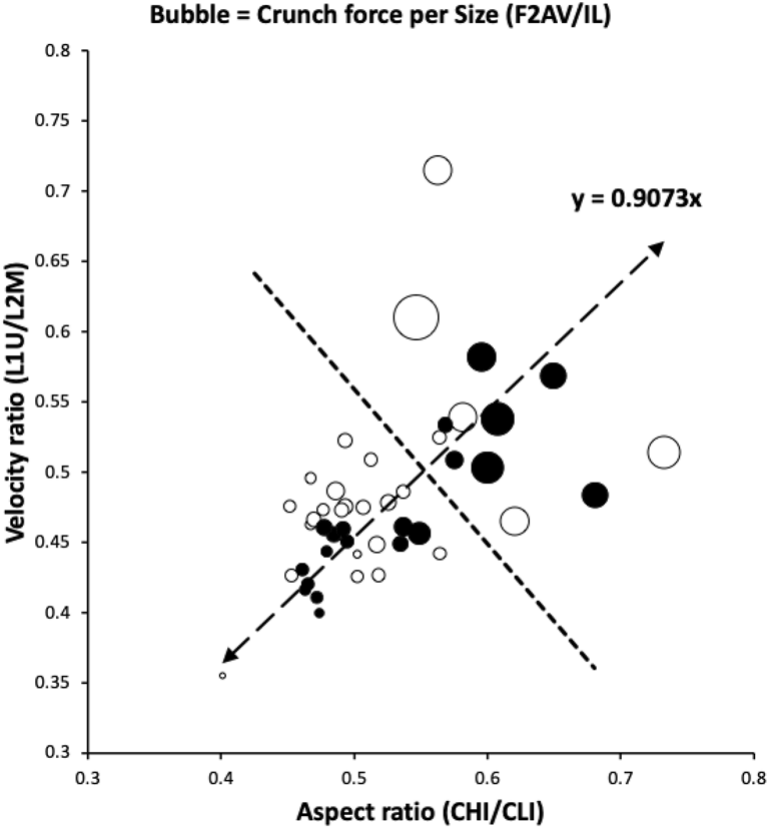
Geometric similarity amongst astigmatans (amended from Bowman 2021 with permission, Creative Commons Attribution 4.0 International License http://creativecommons.org/licenses/by/4.0/). Black circles = species common in bird nests (Table 1). Note clustering around almost 1:1 regression line. Populations *Aleuroglyphus ovatus* AL2, *Chortoglyphus arcuatus* CH1, *Dermatophagoides pteronyssinus* D3, *Glycometrus hugheseae* G3, *Lepidoglyphus destructor* G6 (far right), S5 and TH3 are above the $SE \leftrightarrow NW$ dashed bisecting line

The free-living saprophagous bird nest community is an astigmatan set showing broad geometric similarity in their cheliceral morphology (bar *Lepidoglyphus destructor* G6 - Fig. 3, Bowman 2021). Large scale culture samples of many astigmatans are available to examine in detail. Geometric similarity of feeding structures has more to do with muscle power and breakage safety margins than any simple scaling by body size or weight on their own (Norberg and Wetterholm-Aldrin 2010).

The performance of an organism in its environment frequently depends more on its composite phenotype than on any particular individual phenotypic traits (Yuan et al. 2019). Accordingly how the actual chelal digit is designed to work mechanically as a whole device (or ‘tool’) for the astigmatan will matter.

Bowman (2024b) showed for the honeybee hive astigmatan interstitial community (*Carpoglyphus lactis*, *Glycyphagus domesticus* and *Tyrophagus putrescentiae*) that despite this overall ‘shrunken-swollen’ geometric similarity, the details of the moveable digit profile and thus how the digit is used as a tool or ‘weapon’ during feeding varied. In particular, three modalities ($\equiv$ functional groups) were delineated: the macro-saprophagous glycyphagid omnivore employed a saw-like design, the fragmentary feeder acarid showed a microsaprophagous specialised stripping design, and the microsaprophagous carpoglyphid possessed a specialist derived slicing design.

This first paper investigating the comparative chelal design of free-living astigmatans in bird nests will (like Bowman 2023a, 2024b) focus on overall moveable digit profile changes introducing a useful transecting and contrasting method to acarine evolutionary morphologists. For some of its detailed arguments it will use freely available material from weapons experts (e.g., Association of Renaissance Martial Arts https://www.thearma.org and Turner 2002). Acarologists are encouraged to consult their material for more insights as to how devices work.

## Materials and methods

Preserved samples of mite individuals from previous work (e.g., Bowman 2021) were used. Mites were cleared in lactic acid and examined as wet mounts with differential interference contrast microscopy (also known as Nomarski interference contrast or Nomarski microscopy, https://en.wikipedia.org/wiki/Differential_interference_contrast_microscopy). Drawings with micrometer scales were made using a Zeiss research microscope drawing tube. Twenty adult female specimens were used of each bird nest inhabiting taxon for locating the 18 projected profile data points to ensure the full rank of matrices and comparison to earlier work. The taxa reviewed cover the four feeding habit dimensions of ‘Surface-living’ versus ‘Interstitial’ by ‘Omnivore’ versus ‘Fragmentary feeder’ (Bowman 2021). No mites from the family Histiostomatidae (or anoetids) were used. Samples of *Austroglycyphagus* species, *Blomia* spp., *Campephilocoptes* spp., *Carpoglyphus nidicolous*, *Dermacarus pilitarsus*, *Dermatophagoides evansi*, *Echimyopus orphanus*, *Euglycyphagus intercalatus*, *Fusacarus* spp., *Glycyphagodes spheniscicola*, *Glycyphagus ornatus*, *Gymnoglyphus longior*, *Hirstia chelidonis*, *Neoxenoryctes reticulatus*, *Psyl!oglyphus parapsyl!us* (Winterschmidtiidae), *Sapracarus tuberculatus*, *Schwiebia talpa*, *Tyrophagus mixtus*, *Tyrophagus formicetorum* and *Mycetoglyphus fungivorus* (also known from bird’s nests) were not available.

**Fig. 4:**
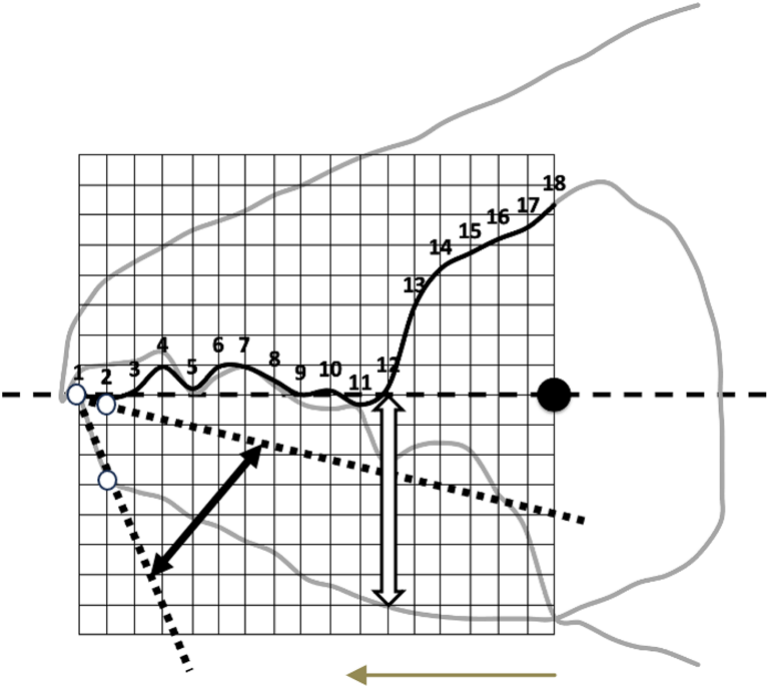
Estimating digit ’tip angle’ (amended from Fig. 6 in Bowman 2024a with permission, Creative Commons Attribution 4.0 International License http://creativecommons.org/licenses/by/4.0/). White double-headed arrow indicates digit depth (*W*, from *L*2*M* axis to ventral surface) at notional end of mastication surface $x_{i_{e}}$ ( = last crossing of *L*2*M* axis at the rise of ascending ramus). Moveable digit tip angle (indicated by double-headed arrowed black line), between extrapolated dotted line joining position of projected locations 1 (tip) and 2 (open circles) with that extrapolated dotted line joining the tip to the moveable digit ventral profile directly below projected location 2 (open circle)

**Fig. 5:**
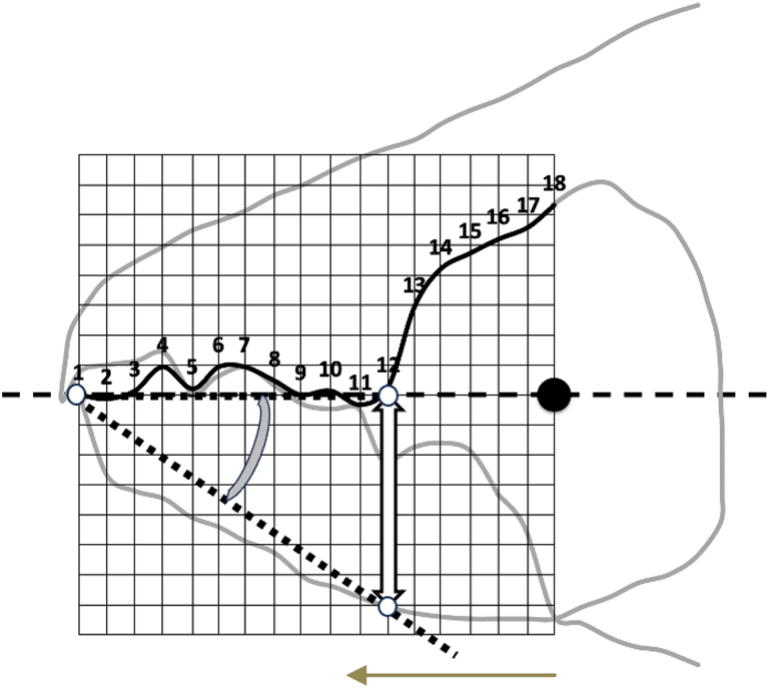
Estimating ’distal digit angle’ (amended from Fig. 6 in Bowman 2024a, Creative Commons Attribution 4.0 International License http://creativecommons.org/licenses/by/4.0/). White double-headed arrow indicates digit depth (*W*, from *L*2*M* axis to ventral surface) at notional end of mastication surface $x_{i_{e}}$ ( = last crossing of *L*2*M* axis at the rise of ascending ramus). Moveable digit distal angle (indicated by grey arc, between extrapolated dotted line joining position of projected locations 1 (tip) and 2 (open circle) on mastication surface and the tip with directly below on the ventral surface of the digit

Measurements for each individual were made for structures outlined in Bowman (2021, 2023a, 2024a, 2024b): *L*2*M*, *L*1*U*, *VR*, Kerf, $\alpha$, the posterior end of the tooth row ($x_{i_{e}}$), digit tip angle (Fig. 4) and digit distal angle (Fig. 5).

The whole profile methods used were those of Bowman (2023a, 2024b).

All data manipulations were carried out in ‘Excel for Mac’ version 16.78.3 and R version 4.3.1 (2023-0616) ‘Beagle Scouts’. Appropriate reduced subsets of measurements were used in analyses if some parameters were linearly dependent. The Riemannian space in which the *av*(*SSCP*) matrices of the projected locations of the moveable digit profiles sit ($\Omega _{i}$ where $i=1...no.\ of\ samples\ (N)$, Bowman 2024b) is decomposed as in the Explanatory Appendix.

For the TRL4 ‘validation in the lab’ of the algorithm (in the Explanatory Appendix), distances used ‘distcov’ and matrix interpolates used ‘estcov’ from the R library ‘shapes’ Dryden (2021). *dsigma* was defined as the average of the within *Tyrophagus palmarum* and the within *Tyrophagus similis* inter-sample distances (following the consensus approach of Bowman 2024a).

If necessary, profile data was prepared using wrap.spd, then: pairwise distances on the semi=positive definite matrix (SPD) manifold used spd.pdist, multidimensional scaling used riem.mds, principal geodesic analysis used riem.pga, geodesic comparisons used riem.distlp, and agglomerative hierarchical clustering used riem.hclust, all from the R package Riemann (0.1.4).

The calculation of consensus $\Omega$ matrices and critical regions follow Bowman (2024b).

### Horizontal ramus features (apparent asperities)

The features of the horizontal ramus (mastication surface) were explored as follows.

As in Bowman (2024a, 2024b) it is illuminating to examine the velocity ($g_{i}$) (and thus the acceleration, $c_{i}$) of moveable digit heights ($y_{i}$) projected onto standard positions on a common *L*2*M* axis. Indeed, the moveable digit projected profile ($[x_{i},y_{i}]$, $i=1...18$) can be usefully summarised (in terms of the angle in radians of its slope $\phi _{i}$). Here $\phi _{i}=tan^{-1}(\frac{y_{i}-y_{i-1}}{x_{i}-x_{i-1}}) i=2...18$. Using the flexure of the mastication surface illustrated in Fig. 7 of Bowman (2024a), this is $\equiv tan^{-1}(f_{i})$.

Converting such values (in radians) to degrees ($^{\circ }$) by multiplication with $\frac{360}{2.\Pi }$ gives: if the increment of the profile at that location rises posteriorly $\Rightarrow$ a $\phi _{i}$ value that is positive, if the increment of the profile at that location falls posteriorly $\Rightarrow$ a $\phi _{i}$ value that is negative. Steeper slopes give larger angles (up to but not including the impossible discontinuity at $\pm 90^{\circ }$). A flat profile increment is equivalent to $\phi =0^{\circ }$. This gives a 17 dimensional hyper-toroidal variable dataset for each individual mite studied which can be examined with statistical contrasts and comparisons between species made.

Dimensional reduction to simpler essential forms is sought whereby perhaps different regions (along *L*2*M*) can be defined by patterns of covariation. This is achieved by one posing a variety of summarising statistical contrasts covering (just) the tooth row (i.e., up to and including $x_{i=10}$ in this study). Each contrast will yield a single scalar value by multiplying the coefficients with their corresponding $\phi _{i}$ value and adding them all up.

Now, one expects for a moveable digit that the surface will either rise, fall or stay level from $y_{1}=0$ at the tip (irrespective of any digit’s depth overall increase for strengthening, Bowman 2024b). This could be tested with a linear contrast of $\phi _{i}$ values (but the latter is not greatly informative of differentiated horizontal ramus features). Rather, a quadratic contrast in transformed $g_{i}$ slopes (thus mapping to a cubic change in $y_{i}$) will detect a non-linear change in the angle of the slope $\phi _{i}$ (i.e., a variable acceleration $c_{i}$) and will indicate a likely apparent tooth or gullet.

Of course the feature or ‘module’ of interest can be of different sizes, so for the first nine $\phi _{i}$ values from the moveable digit tip over all individuals and taxa the following row of coefficients

$$\left( \begin{array}{ccccccccc} 1,1,1,-2,-2,-2,1,1,1 \end{array} \right)$$

tests for overall ‘breasting’ of the mastication surface (i.e. a single differentiated feature covering the whole surface). These coefficients could be considered as ‘winding numbers’ for analysing phase angles on a hypertorus. In this case the coefficients should be rescaled by multiplication by $\frac{1}{\sqrt{18}}$ for comparability with other hypotheses. This contrast is labeled contrast *a*.

Alternatively as ‘toothiness’ ie expected to be local, four modules can be tested for using the rows of

$$\left( \begin{array}{ccccccccc} 1 & 1 & -2 & -2 & 1 & 1 & 0 & 0 & 0\\ 0 & 1 & 1 & -2 & -2 & 1 & 1 & 0 & 0\\ 0 & 0 & 1 & 1 & -2 & -2 & 1 & 1 & 0\\ 0 & 0 & 0 & 1 & 1 & -2 & -2 & 1 & 1\\ \end{array} \right)$$

scaled by $\frac{1}{\sqrt{12}}$ for comparability with the other hypotheses. This offers different mastication locations for a six position differentiated feature. These are labelled contrasts *b*1, *b*2, *b*3, *b*4 and divide the mastication surface into four regions, distally $\rightarrow$ proximally to the condyle.

Finally, seven rows of coefficients (scaled by $\frac{1}{\sqrt{6}}$) from

$$\left( \begin{array}{ccccccccc} 1 & -2 & 1 & 0 & 0 & 0 & 0 & 0 & 0\\ 0 & 1 & -2 & 1 & 0 & 0 & 0 & 0 & 0\\ 0 & 0 & 1 & -2 & 1 & 0 & 0 & 0 & 0\\ 0 & 0 & 0 & 1 & -2 & 1 & 0 & 0 & 0\\ 0 & 0 & 0 & 0 & 1 & -2 & 1 & 0 & 0\\ 0 & 0 & 0 & 0 & 0 & 1 & -2 & 1 & 0\\ 0 & 0 & 0 & 0 & 0 & 0 & 1 & -2 & 1\\ \end{array} \right)$$

will test ‘atomic’ dental features (and thus two independent atomic modules being simultaneously present in a mastication surface when the rows are orthogonal to each other). These are labelled contrasts *c*1, *c*2, *c*3, *c*4, *c*5, *c*6, *c*7 and divide the mastication surface into seven features, distally $\rightarrow$ proximally to the condyle. Such contrasts detect the apparent peaks and the apparent gullets across a regularly spaced sampled moveable digit profile.

Note that the quadratic contrast of slope automatically allows for asymmetric apparent asperities, irrespective of whether the module is an apparent peak or gullet. Also note that these are for $g_{i}$ values i.e., the *i* index is the location index for the measured $[x_{i},y_{i}]$ is one less. This means that as the moveable digit tip is a fixed point at the $x_{1}=0$ origin, the twelve contrasts *a*1, *b*1....*b*4, *c*1...*c*7 are each ‘centred’ at the equivalent of locations along the reference *L*2*M* axis of $x_{6},x_{4.5},x_{5.5},x_{6.5},x_{7.5},x_{3},x_{4},x_{5},x_{6},x_{7},x_{8},x_{9}$ respectively (using the notation of Bowman 2024a). Finally, note that a negative value for the scalar indicates an apparent ‘peak’ and a positive value indicates an apparent ’gullet’.

In every case one should visually check that the projected locations used in the contrast are reasonable to summarise or test that particular feature across all individuals and all taxa within any comparison (as the mastication surface length *m* can vary between species).

## Results

### General observations

**Fig. 6:**
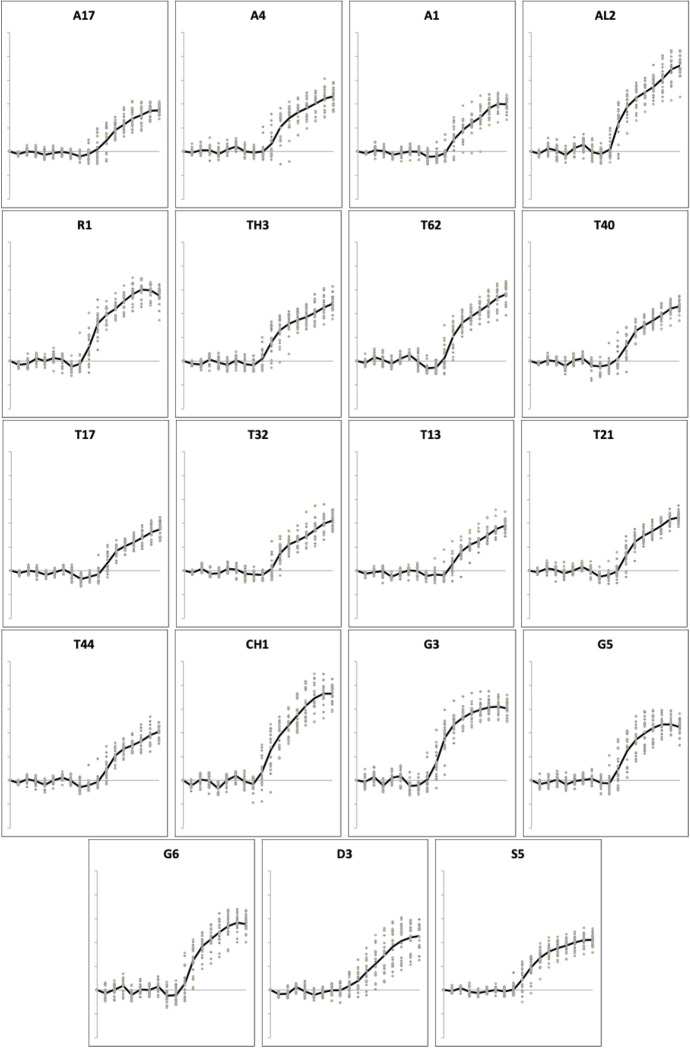
Profiles scaled to equivalent *L*2*M* reference axis in the same order as Table 1. Acaridae: *Acarus farris* A17, *Acarus gracilis* A4, *Acarus immobilis* A1, *Aleuroglyphus ovatus* AL2, *Rhizoglyphus robini* R1, *Thyreophagus entomophagus* TH3, *Tyrolichus casei* T62, *Tyrophagus longior* T40, *Tyrophagus palmarum* T17, T32, *Tyrophagus putrescentiae* T13, *Tyrophagus similis* T21, T44. Chortoglyphidae: *Chortoglyphus arcuatus* CH1. Glycyphagidae: *Glycometrus hugheseae* G3, *Glycyphagus domesticus* G5, *Lepidoglyphus destructor* G6. Pyroglyphidae: *Dermatophagoides pteronyssinus* D3. Suidasidae: *Suidasia pontifica* S5. Solid line = mean profile for each taxon

The measured profiles of the moveable digit from each species (scaled to equivalent *L*2*M* reference axis size) are shown in Fig. 6.

**Fig. 7:**
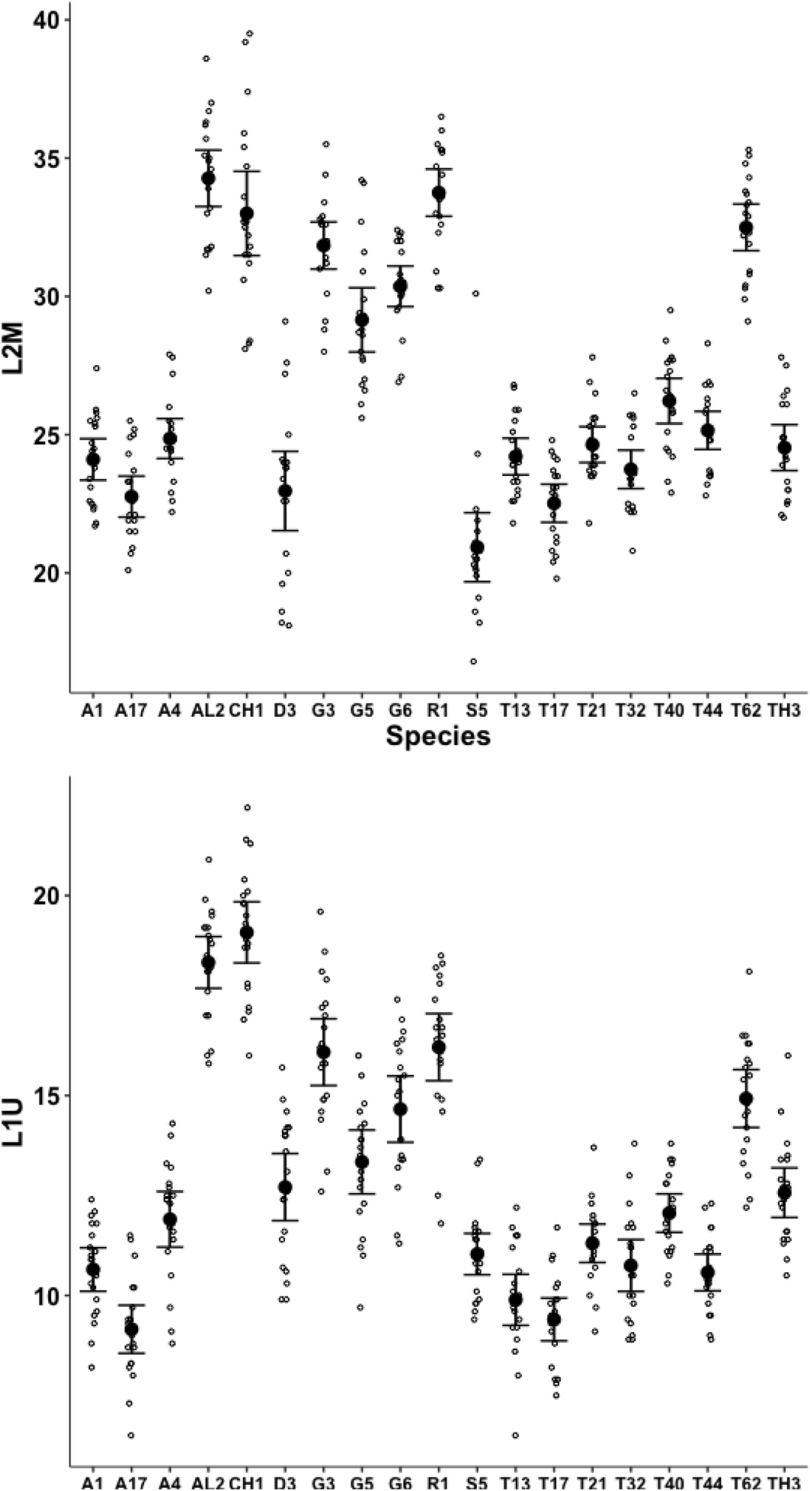
Upper: observed data (small open dots) with mean (large black dot) and $95\%$ confidence intervals (as ’T’ bars) by species (in alphanumeric order) for output moment lever arm (reference axis) *L*2*M* in $\mu m$. Lower: Observed data (small open dots) with mean (large black dot) and $95\%$ confidence intervals (as ’T’ bars) by species (in alphanumeric order) for input moment lever arm *L*1*U* in $\mu m$

**Fig. 8:**
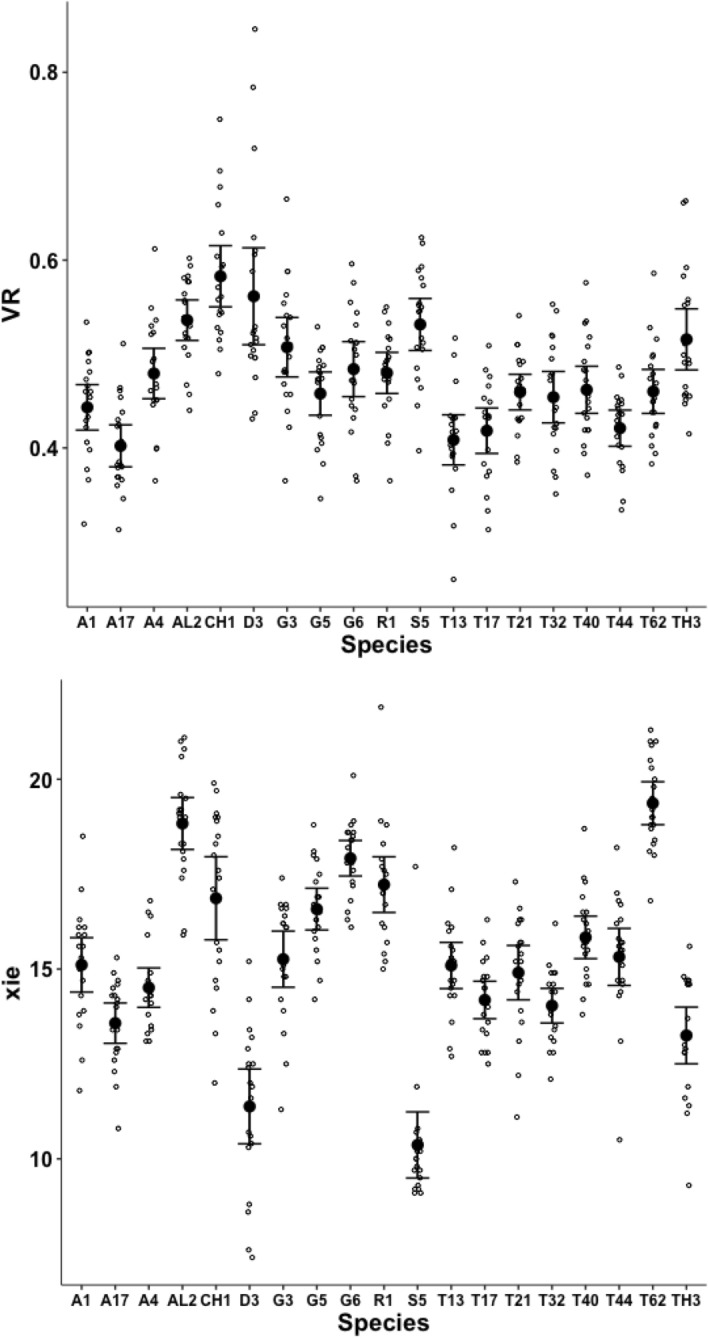
Upper: Observed data (small open dots) with mean (large black dot) and $95\%$ confidence intervals (as ’T’ bars) by species (in alphanumeric order) for chelal velocity ratio (*VR*). Lower: Observed data (small open dots) with mean (large black dot) and $95\%$ confidence intervals (as ’T’ bars) by species (in alphanumeric order) for the end of mastication surface (*e*) distance along L2M axis towards condyle ($x_{i_{e}}$) in $\mu m$

Measurements for each individual made for the chelal parameters in Bowman (2021): *L*2*M*, *L*1*U* and *VR* are summarised in Figs. 7 and 8.

A two-way ANOVA shows that the chelae in the individuals representing each species in this study do not differ significantly from the design of those previously measured in Bowman (2021). While species were found to be different as expected, no interaction terms were significant. Testing the main effect of ‘old’ (2021) measurements versus ’new’ measurements (in this paper) gave: *L*2*M* $F_{1,722}=3.319$ ns; *L*1*U* $F_{1,722}=3.237$ ns; *VR* $F_{1,722}=0.281$ ns. Accordingly, the chelal categorisations in Bowman (2021) are assumed to still apply.

**Fig. 9:**
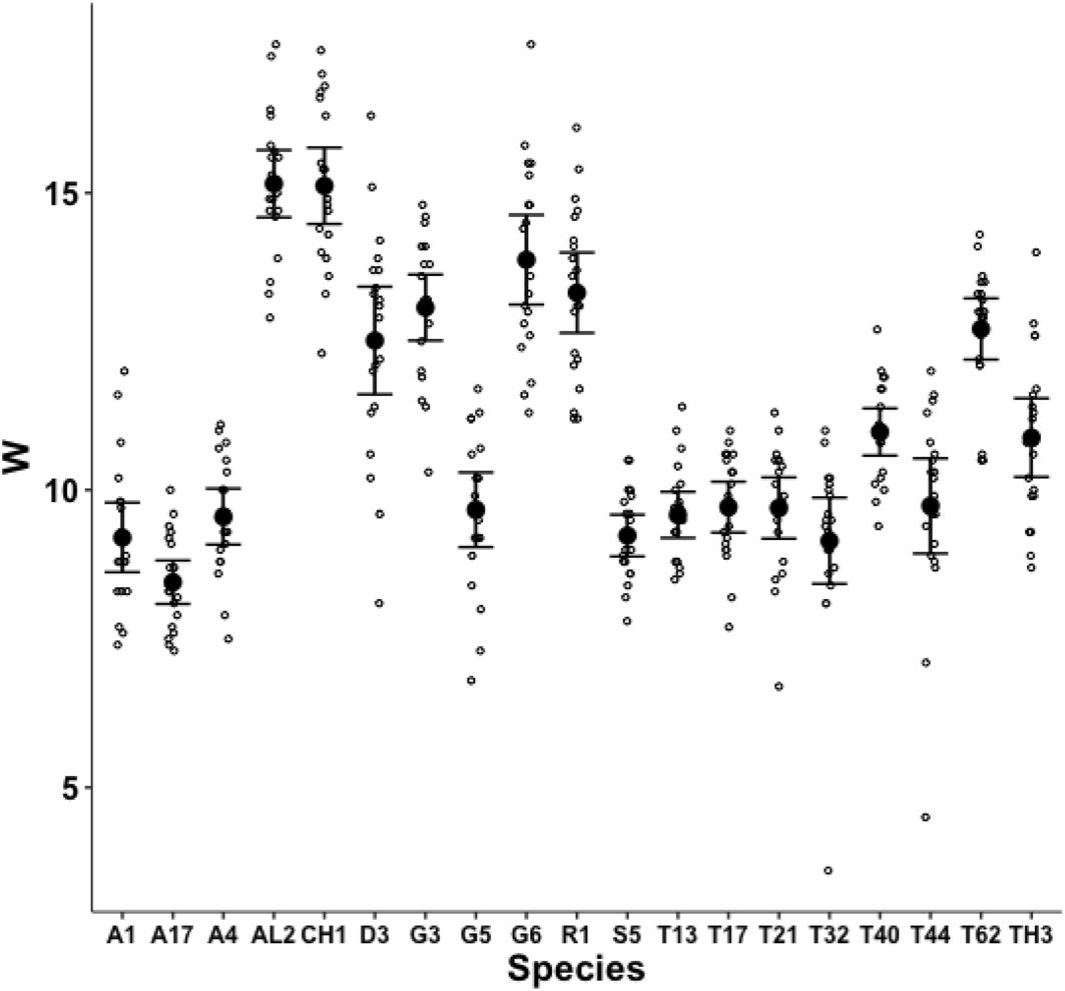
Observed data (small open dots) with mean (large black dot) and $95\%$ confidence intervals (as ’T’ bars) by species (in alphanumeric order) for the ’saw width’ W in $\mu m$

**Fig. 10:**
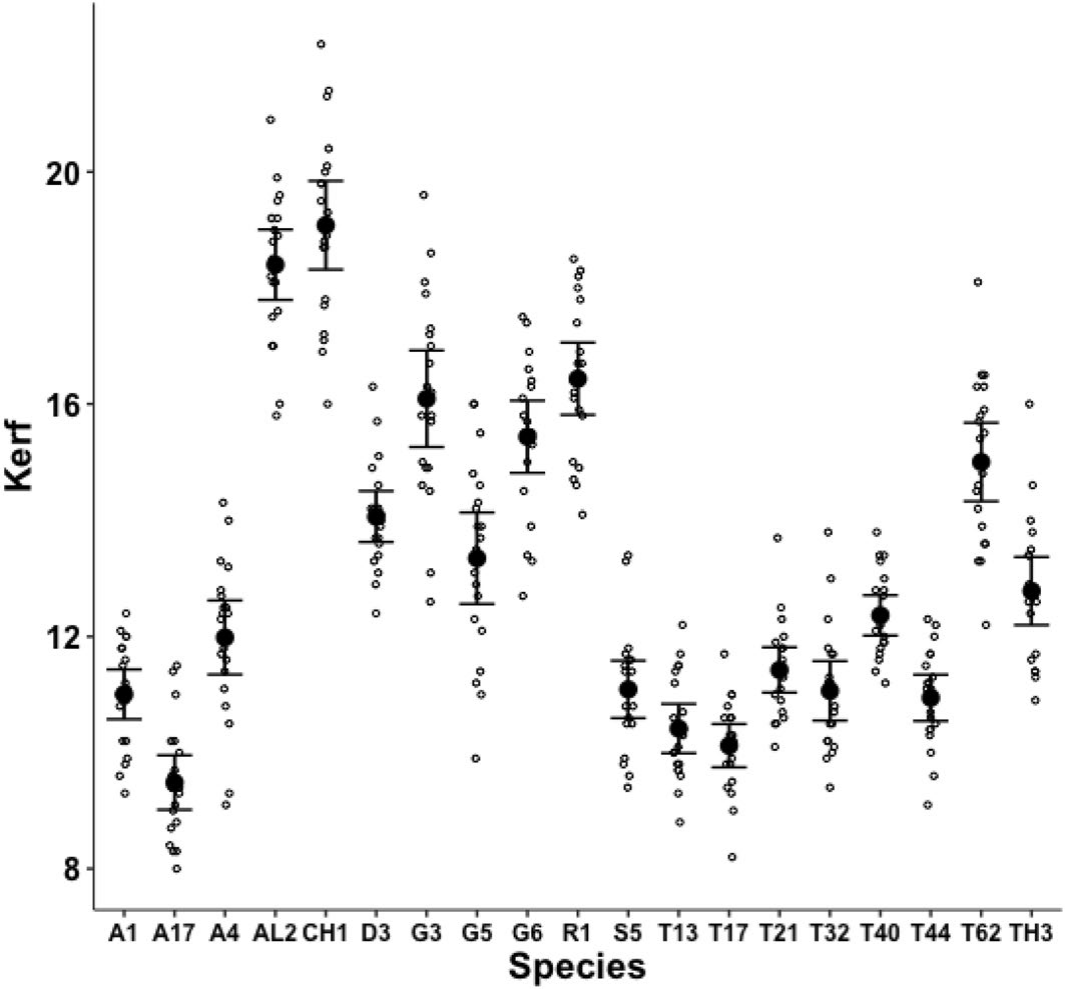
Observed data (small open dots) with mean (large black dot) and $95\%$ confidence intervals (as ’T’ bars) by species (in alphanumeric order) for the size of the Kerf in $\mu m$

**Fig. 11:**
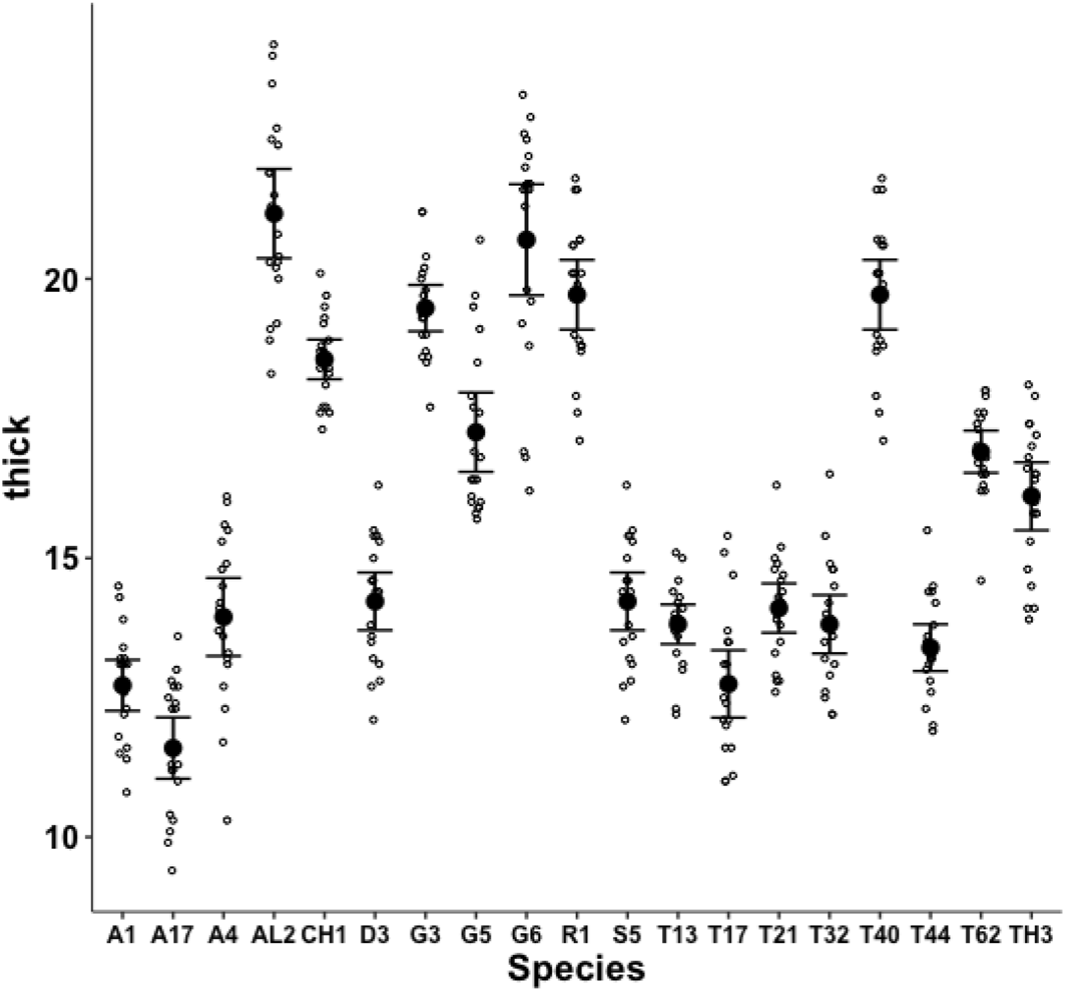
Observed data (small open dots) with mean (large black dot) and $95\%$ confidence intervals (as ’T’ bars) by species (in alphanumeric order) for the moveable digit measure *thick* in $\mu m$

**Fig. 12:**
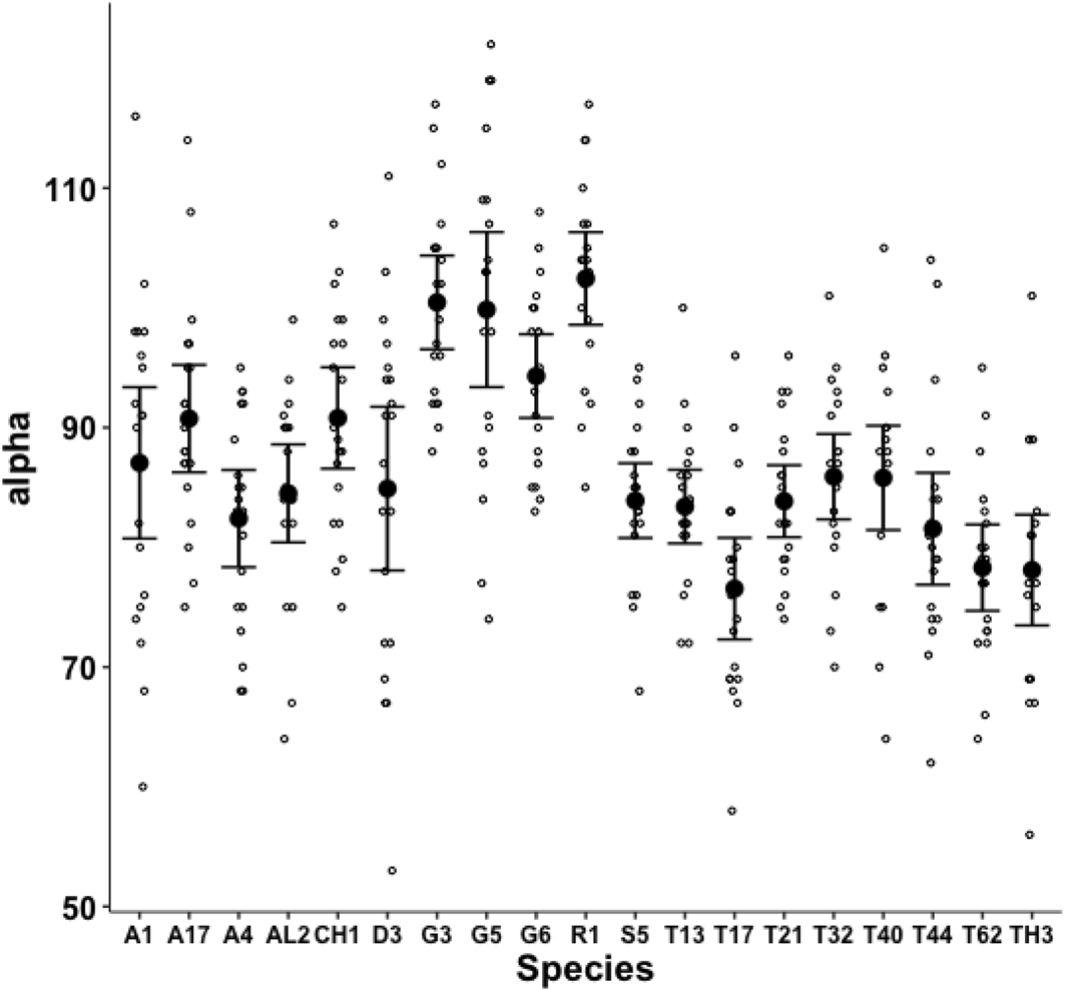
Observed data (small open dots) with mean (large black dot) and $95\%$ confidence intervals (as ’T’ bars) by species (in alphanumeric order) for the moveable digit design $\alpha$ in $^{\circ }$

**Fig. 13:**
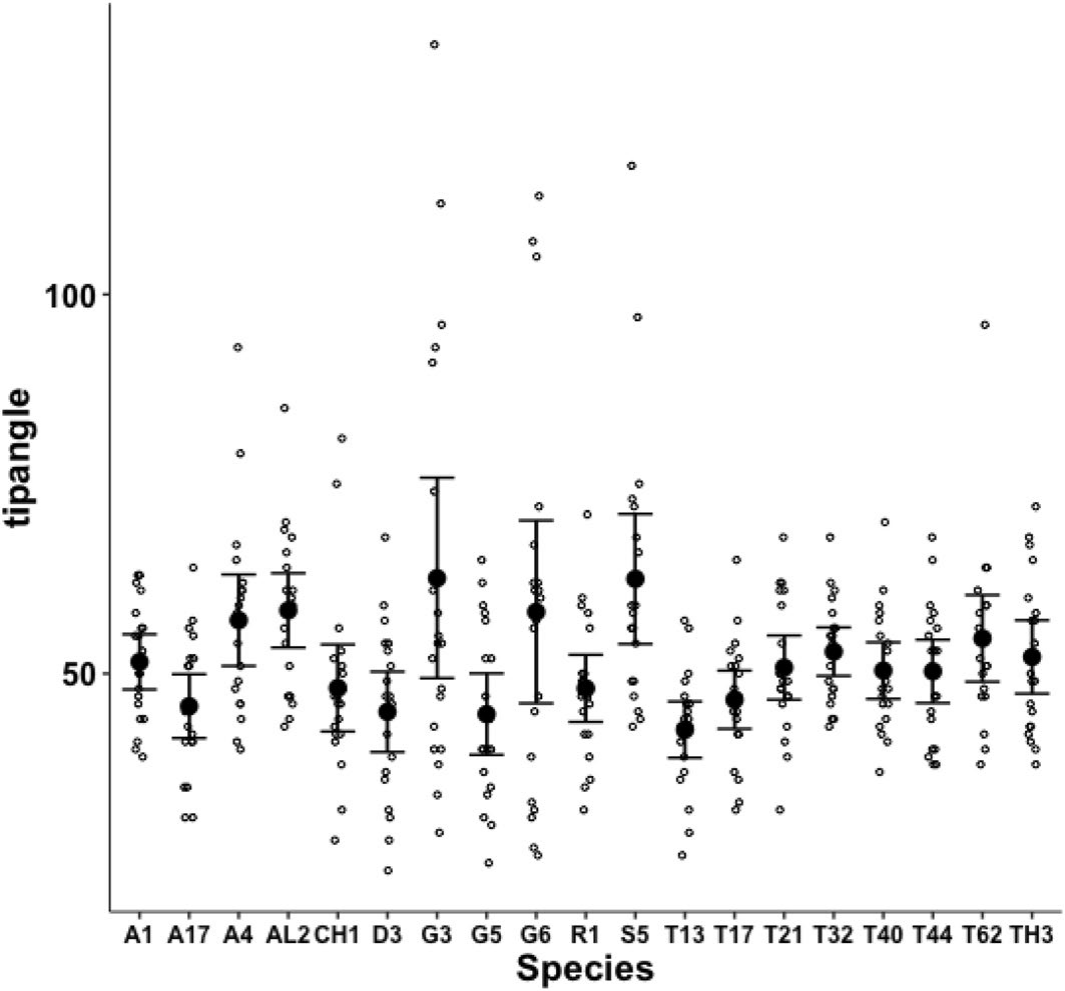
Observed data (small open dots) with mean (large black dot) and $95\%$ confidence intervals (as ’T’ bars) by species (in alphanumeric order) for the moveable digit tip angle in $^{\circ }$

**Fig. 14:**
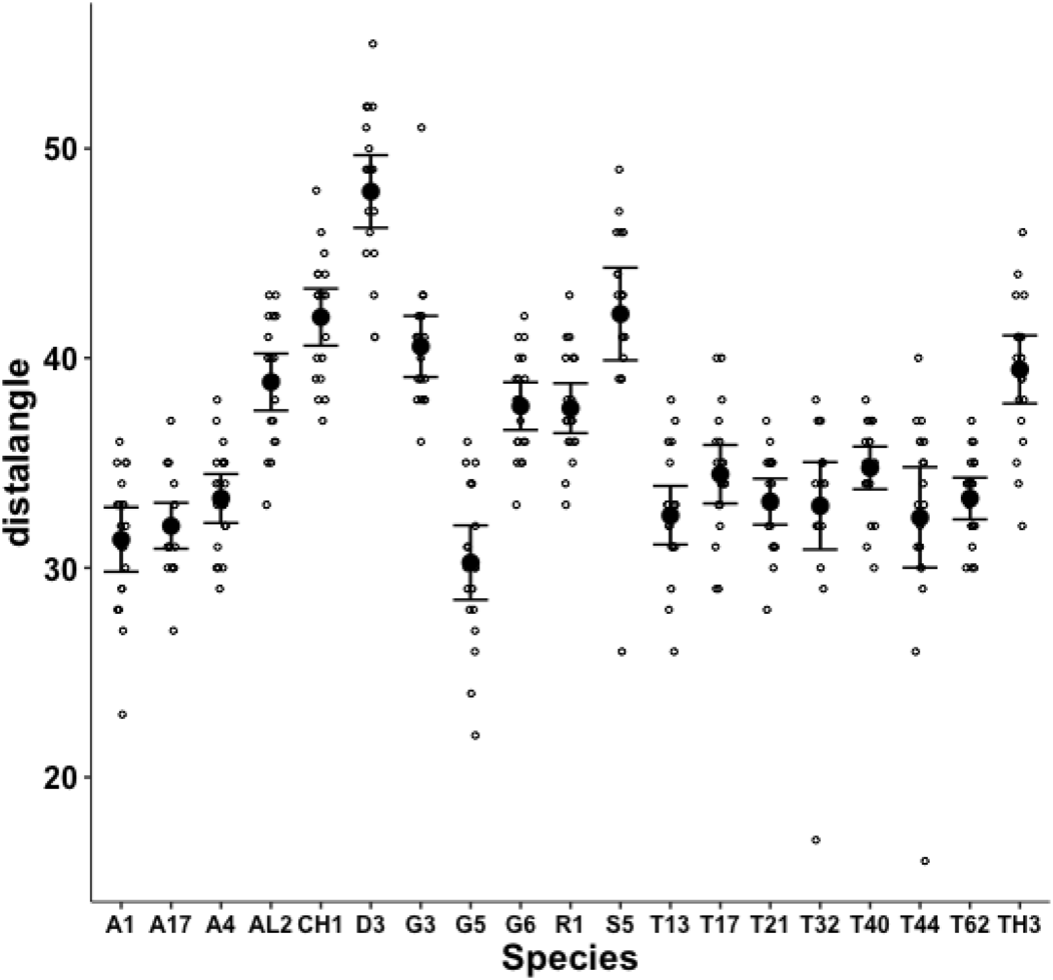
Observed data (small open dots) with mean (large black dot) and $95\%$ confidence intervals (as ’T’ bars) by species (in alphanumeric order) for the moveable digit distal angle in $^{\circ }$

Measurements for each individual made for parameters from Bowman (2024a): the posterior end of the tooth row ($x_{i_{e}}$), ‘saw width’ *W*, Kerf, *thick*, $\alpha$, tip angle and distal digit angle are summarised in Figs. 8, 9, 10, 11, 12, 13 and 14.

A significant effect of species for the end of the mastication surface ($x_{i_{e}}$) was detected ($F_{18,361}=47.74, p<0.001$) with both long length and short length tooth row species being found (Fig. 8). However, $x_{i_{e}}$ values scale well with *L*2*M* across species (regression through zero $R^2=0.993$, *plot not shown*) indicating a simple overall size scaling factor between the bird nest astigmatans rather than trophic differentiation *per se*.

A significant effect of species for depth of moveable digit at the end of the mastication surface (*W*) was detected ($F_{18,361}=58.03, p<0.001$, Fig. 9). However, as expected (since the ‘saw depth’ *W* is the moveable digit depth at approximately $VR_{i_{_e}}=1$, Bowman 2024b) the observed values scale well with the adductive force *F*1*AV* along the closing tendon across the species (regression through zero $R^2=0.966$, *plot not shown*). The bird nest astigmatan chelae are simply appropriately strengthened as expected.

A significant effect of species for the size of the Kerf ($\equiv$ sawn groove width) was detected ($F_{18,361}=109.8, p<0.001$) suggesting ‘full-kerf’ (e.g., *Aleuroglyphus ovatus* AL2 and *Chortoglyphus arcuatus* CH1) and particularly ‘thin-kerf’ (e.g., *Acarus farris* A17) variants of the astigmatan moveable digit exist when used as a saw (Fig. 10). A significant effect of species for the moveable digit measure *thick* was detected ($F_{18,361}=121.9, p<0.001$, Fig. 11). However, *thick* predicts the size of the Kerf well (regression through zero $R^2=0.988$, *plot not shown*) suggesting that they are all indicating the same underlying trophic distinction (of digit robustness).

Although over all the astigmatans the $\alpha$ design angle (see Akimov and Gaichenko 1976) is around $90^{\circ }$ (Fig. 12), a significant effect of species was detected ($F_{18,361}=12.62, p<0.001$). *Glycometrus hugheseae* G3, *Glycyphagus domesticus* G5, *Lepidoglyphus destructor* G6 and *Rhizoglyphus robini* R1 have elevated values compared to the acarids. The Discussion section below explains the significance of this.

Despite Fig. 13 showing considerable overlap in data for the digit tip angle of each species (with an overall average $\approx 52^{\circ }$), a significant effect of species was detected ($F_{18,361}=3.949, p<0.001$). *Glycometrus hugheseae* G3, *Lepidoglyphus destructor* G6 and *Suidasia pontifica* S5 have seemingly higher values on average (although this may be due to some high outliers driven by an initial rise in digit profile rather than a fall posterior of the digit tip in some individuals’ slide preparations).

A significant effect of species for moveable digit distal angle was detected ($F_{18,361}=41.15, p<0.001$, Fig. 14). As expected this measurement is well correlated with both the digit tip angle ($R^2=0.9793$, *plot not shown*) and the ‘saw depth’ *W* ($R^2= 0.9776$, *plot not shown*). This represents structural investment distally and proximally (to the condyle) respectively. Larger mites and those with greater output adductive force (*F*2*AV*) have larger digit distal angle values on average ($R^2=0.8974$ and $R^2=0.9450$ respectively, *plots not shown*) commensurate with the required robustification.

### Moveable digit profiles as a whole

As in Bowman (2024a, 2024b) it is illuminating to examine the moveable digit heights ($y_{i}$) projected onto standard positions on a common *L*2*M* axis for any morphological transitions across species. Geodesic paths between species can be examined by the TRANSECT algorithm (see Explanatory Appendix).

**Fig. 15:**
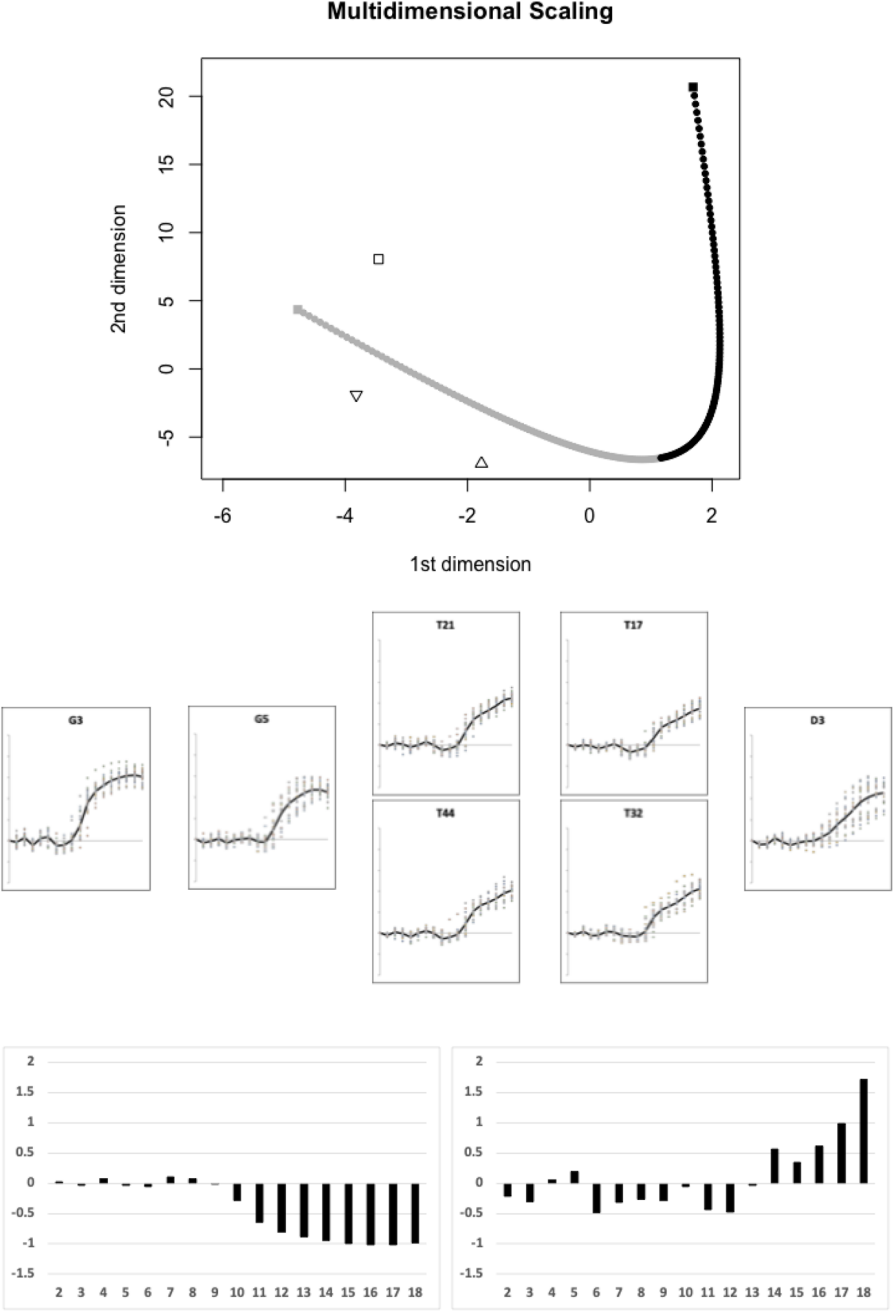
First candidate transitional path through moveable digit $\Omega$ design space of free-living astigmatan mites common in bird nests ($lengeodesic=14.79342$). Upper, two dimensional multidimensional scaling showing transect between *Glycometrus hugheseae* G3 = grey square and *Dermatophagoides pteronyssinus* D3 = black square. Geodesic in dots coloured correspondingly into two halves. Three taxa are within the critical region of $1.645\sigma =8.15864$ and can be considered as being on the same transit: *Tyrophagus palmarum* (T17 & T32 consensus) = pyramid (pointing upwards), *Tyrophagus similis* (T21 & T44 consensus) = triangle (pointing downwards), *Glycyphagus domesticus* G5 = open square, Middle, profiles ordered left to right as per their nearest position along geodesic. Lower, on left first relative eigenvector from $\Omega _{G3}$ rightwards towards $\Omega _{D3}$, on right first relative eigenvector from $\Omega _{D3}$ leftwards towards $\Omega _{G3}$. Numbers 2...18 = measurement positions along moveable digit (1 = tip)

Fig. 15 illustrates the first candidate evolutionary transition between the moveable digit profile $\Omega$ matrix of *Dermatophagoides pteronyssinus* D3 and that of *Glycometrus hugheseae* G3 estimated by the TRANSECT algorithm. three other species effectively sit on this first geodesic (*Tyrophagus palmarum* T17 & T32 consensus, *Tyrophagus similis* T21 & T44 consensus, and *Glycyphagus domesticus* G5.

**Fig. 16:**
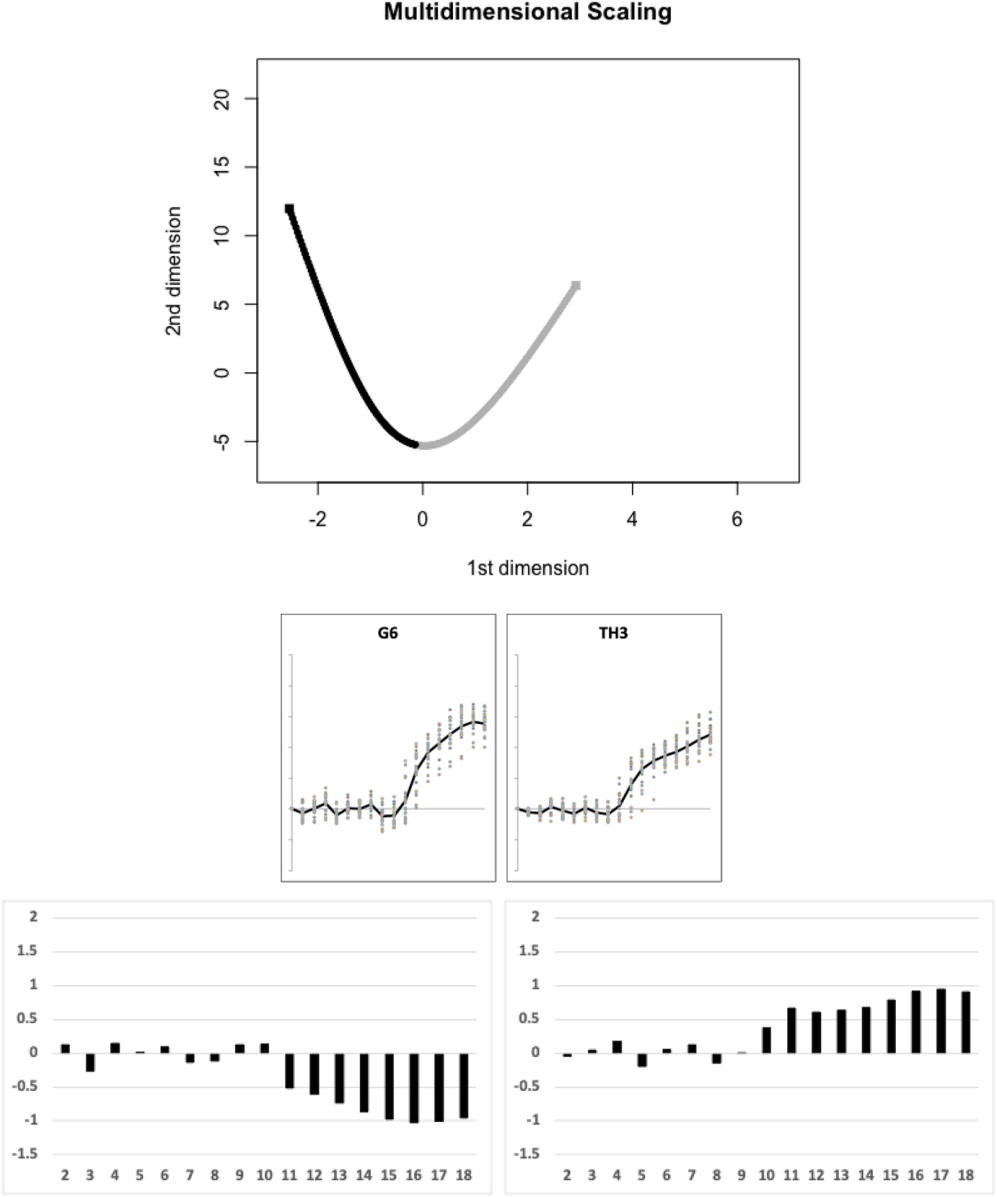
Second candidate transitional path through moveable digit $\Omega$ design space of free-living astigmatan mites common in bird nests ($lengeodesic=13.19223$). Upper, two dimensional multidimensional scaling showing transect between *Thyreophagus entomophagus* TH3 = grey square and *Lepidoglyphus destructor* G6 = black square. Geodesic in dots coloured correspondingly into two halves. No remaining species are within the critical region of $1.645\sigma =8.15864$ and could be considered as being on the same transit: Middle, profiles ordered left to right as per geodesic. Lower, on left first relative eigenvector from $\Omega _{G6}$ rightwards towards $\Omega _{TH3}$, on right first relative eigenvector from $\Omega _{TH3}$ leftwards towards $\Omega _{G6}$. Numbers 2...18 = measurement positions along moveable digit (1 = tip)

Of the remaining species, Fig. 16 illustrates the next (second) candidate evolutionary transition between the moveable digit profile $\Omega$ matrix of *Thyreophagus entomophagus* TH3 and that of *Lepidoglyphus destructor* G6 estimated by the TRANSECT algorithm. No other species effectively sit on this second geodesic.

**Fig. 17:**
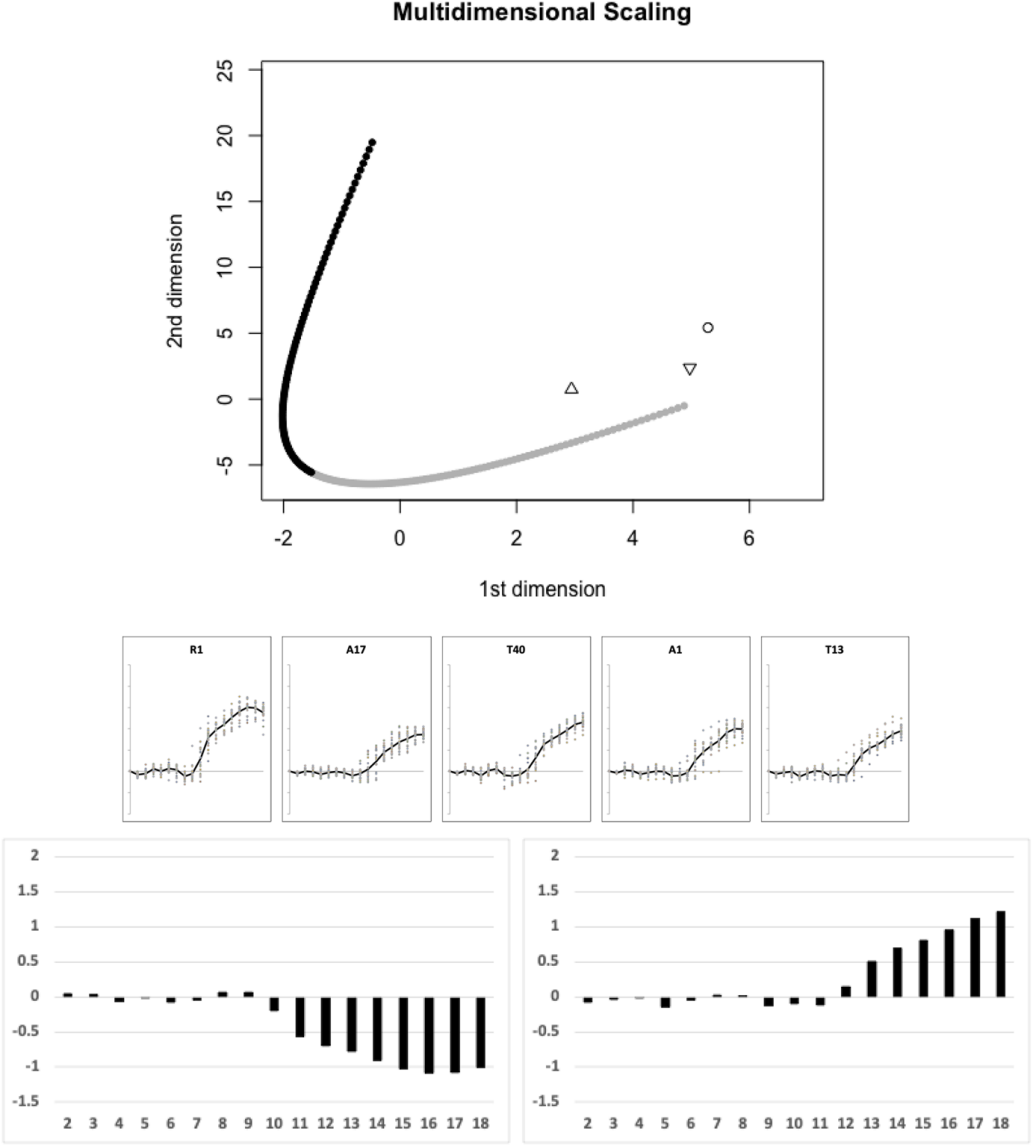
Third candidate transitional path through moveable digit $\Omega$ design space of free-living astigmatan mites common in bird nests ($lengeodesic=12.747022$). Upper, two dimensional multidimensional scaling showing transect between *Rhizoglyphus robini* R1 = black square and *Tyrophagus putrescentiae* T13 = grey square. Geodesic in dots coloured correspondingly into two halves. Three remaining species are within the critical region of $1.645\sigma =8.15864$ and could be considered as being as being on the same transit: *Acarus farris* A17 = pyramid (pointing upwards), *Acarus immobilis* A1 = triangle (pointing downwards), *Tyrophagus longior* T40 = open square, Middle, profiles ordered left to right as per their nearest position along geodesic. Lower, on left first relative eigenvector from $\Omega _{R1}$ rightwards towards $\Omega _{T13}$, on right first relative eigenvector from $\Omega _{T13}$ leftwards towards $\Omega _{R1}$. Numbers 2...18 = measurement positions along moveable digit (1 = tip)

Of the remaining species, Fig. 17 illustrates the next (third) candidate evolutionary transition between the moveable digit profile $\Omega$ matrix of *Rhizoglyphus robini* R1 and that of *Tyrophagus putrescentiae* T13 estimated by the TRANSECT algorithm. *Acarus farris* A17, *Tyrophagus longior* T40 and *Acarus immobilis* A1 effectively sit on this third geodesic.

**Fig. 18:**
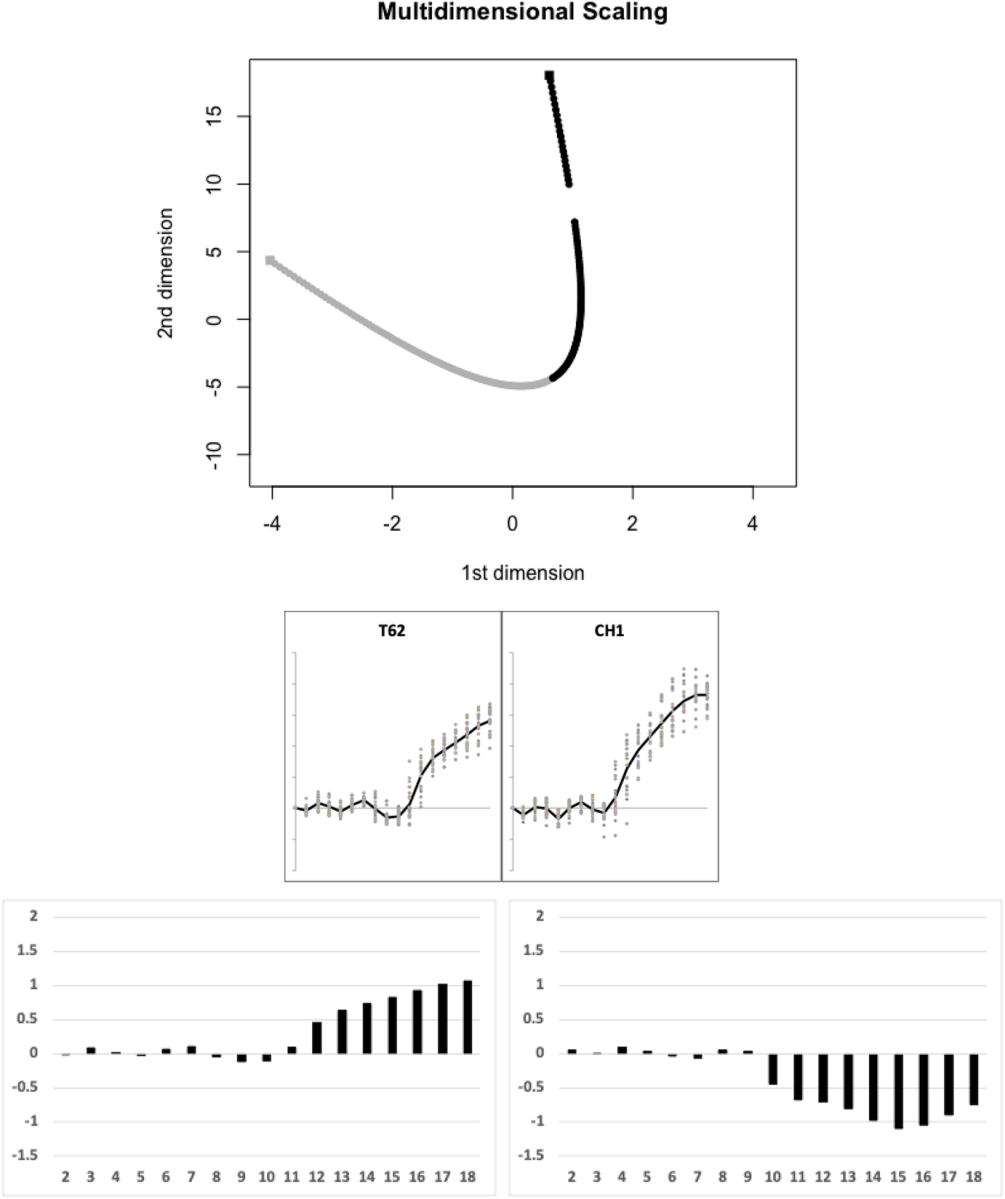
Fourth candidate transitional path through moveable digit $\Omega$ design space of free-living astigmatan mites common in bird nests ($lengeodesic=12.080823$). Upper, two dimensional multidimensional scaling showing transect between *Chortoglyphus arcuatus* CH1 = black square and *Tyrolichus casei* T62 = grey square. Geodesic in dots coloured correspondingly into two halves. Break is due to numerical instabilities in *estcov* and *distcov* routines. No remaining species are within the critical region of $1.645\sigma =8.15864$ and could be considered as being on the same transit: Middle, profiles ordered left to right as per geodesic. Lower, on left first relative eigenvector from $\Omega _{T62}$ rightwards towards $\Omega _{CH1}$, on right first relative eigenvector from $\Omega _{CH1}$ leftwards towards $\Omega _{T62}$. Numbers 2...18 = measurement positions along moveable digit (1 = tip)

Of the remaining species, Fig. 18 illustrates the next (fourth) candidate evolutionary transition between the moveable digit profile $\Omega$ matrix of *Chortoglyphus arcuatus* CH1 and that of *Tyrolichus casei* T62 estimated by the TRANSECT algorithm. No other species effectively sit on this fourth geodesic.

**Fig. 19:**
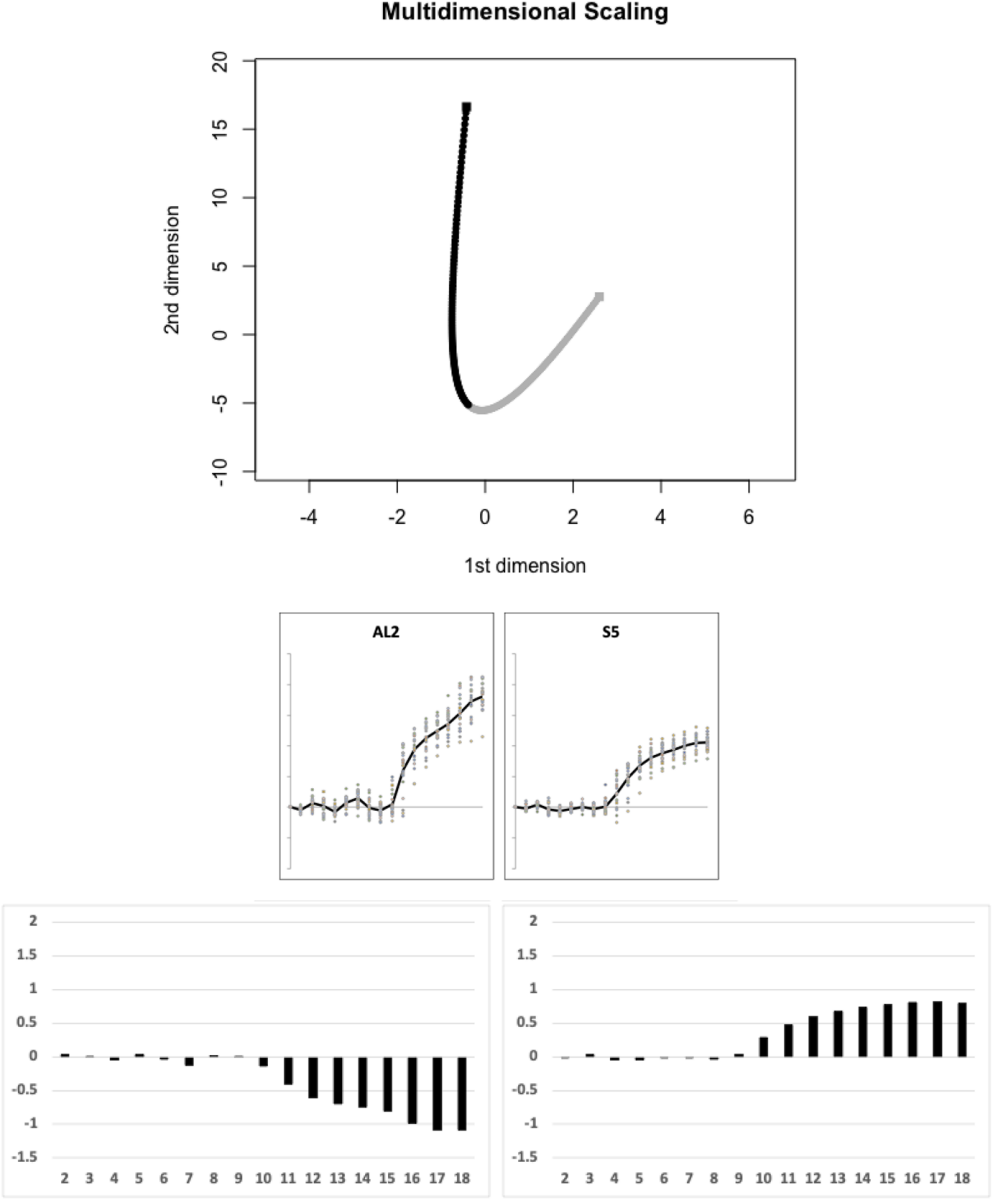
Fifth candidate transitional path through moveable digit $\Omega$ design space of free-living astigmatan mites common in bird nests ($lengeodesic=11.78468$). Upper, two dimensional multidimensional scaling showing transect between *Aleuroglyphus ovatus* AL2 = black square and *Suidasia pontifica* S5 = grey square. Geodesic in dots coloured correspondingly into two halves. No remaining species are within the critical region of $1.645\sigma =8.15864$ and could be considered as being on the same transit: Middle, profiles ordered left to right as per geodesic. Lower, on left first relative eigenvector from $\Omega _{AL2}$ rightwards towards $\Omega _{S5}$, on right first relative eigenvector from $\Omega _{S5}$ leftwards towards $\Omega _{AL2}$. Numbers 2...18 = measurement positions along moveable digit (1 = tip)

Of the remaining species, Fig. 19 illustrates the next (fifth) candidate evolutionary transition between the moveable digit profile $\Omega$ matrix of *Aleuroglyphus ovatus* AL2 and that of *Suidasia pontifica* S5 estimated by the TRANSECT algorithm. No other species effectively sit on this fifth geodesic.

*Acarus gracilis* A4 was left as a singleton species.

Each candidate transit path contrasts a design of a strong ascending and basal ramus (e.g., *Glycometrus hugheseae* G3, *Lepidoglyphus destructor* G6, *Rhizoglyphus robini* R1, *Chortoglyphus arcuatus* CH1 and *Aleuroglyphus ovatus* AL2) with species showing a reduced investment posterior of the horizontal ramus making it into a ‘slab-shape’ (e.g. *Dermatophagoides pteronyssinus* D3, *Thyreophagus entomophagus* TH3, *Tyrophagus putrescentiae* T13, *Tyrolichus casei* T62 and *Suidasia pontifica* S5). *Tyrophagus palmarum*, *Tyrophagus similis*, Four taxa, *Glycyphagus domesticus* G5, *Acarus farris* A17, *Tyrophagus longior* T40 and *Acarus immobilis* A1 appear to be intermediate forms along the different geodesics. Whilst each geodesic is distinct, the general pattern of relative eigenvectors for each is broadly similar.

This overall profile measurement analysis does not appear to detect major distinctions within the horizontal rami. *Tyrophagus palmarum* and *Tyrophagus similis* may be a central ‘non-specialised design‘ in the region where all the transects essentially cross or where they are at least nearby to each other. The latter two species have ‘slab-like’ basal rami. The first candidate evolutionary path runs from glycyphagids through acarids to pyroglyphids. The third candidate evolutionary path features well known agricultural pests (*Rhizoglyphus robini* R1, *Tyrophagus longior* T40, *Tyrophagus putrescentiae* T13). The data cloud looks like a single assembly of ‘prickles’ as on the cactus *Mammillaria balsasoides* or a single areole with multiple spines on the tree-like *Rhodocactus grandifolius* (see https://en.wikipedia.org/wiki/Thorns,_spines,_and_prickles) rather than a multidimensional ball.

**Fig. 20:**
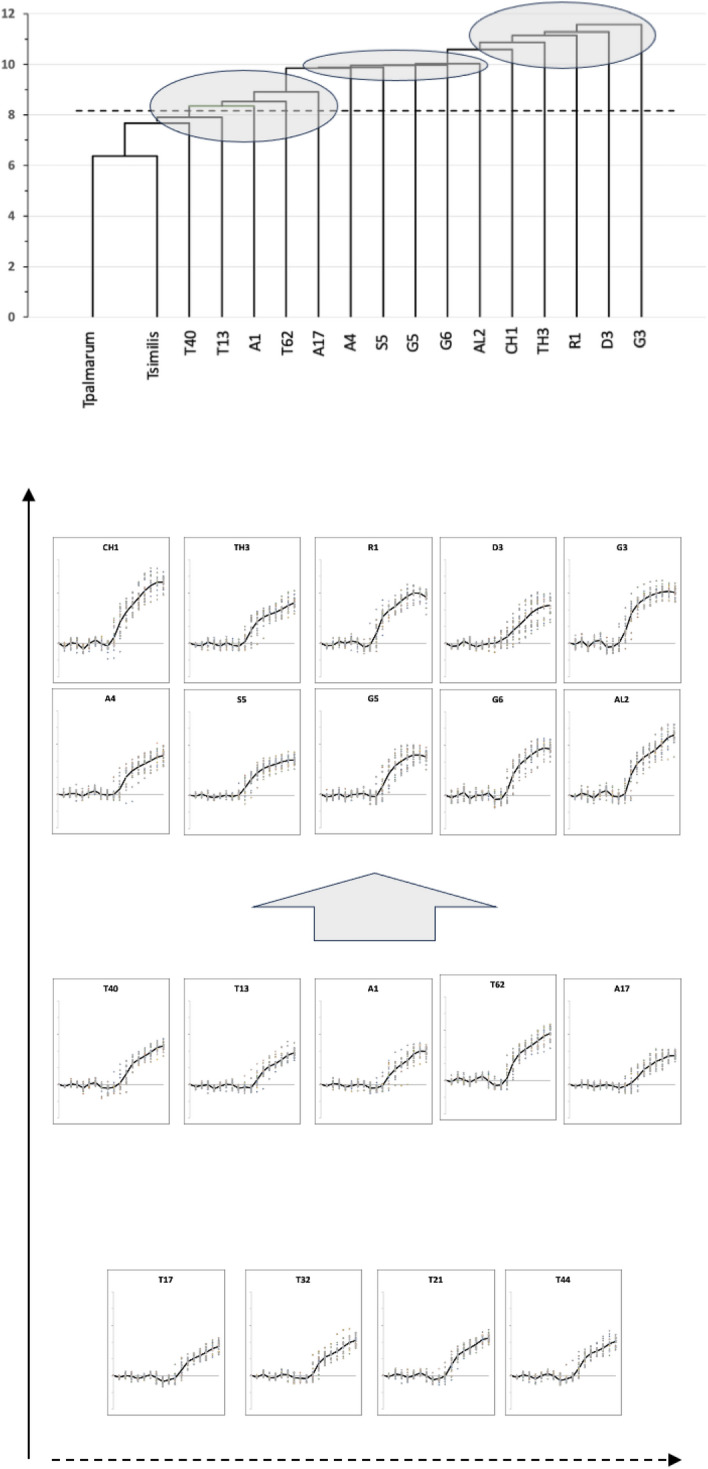
Upper. Single-link hierarchical clustering of the pairwise inter-taxon Riemannian distances for moveable digit data. Dashed line is critical $1.645\sigma =8.15864$ value. Note grey groupings. Lower. Profiles arranged in increasing distance from each other (arrow up the page) and within cluster increasing distance (dashed arrow across the page). Large grey arrow marks significant design change

Indeed a single-link hierarchical clustering of the pairwise inter-taxon Riemannian distances for the moveable digit data (i.e., where the distance between two clusters is the minimum distance between members of the two clusters) shows (Fig. 20) that

- *Tyrophagus palmarum* and *Tyrophagus similis* are indistinguishable from replicates of each other and typify a basal form
- *Tyrophagus longior* T40, *Tyrophagus putrescentiae* T13, *Acarus immobilis* A1, *Tyrolichus casei* T62 and *Acarus farris* A17 are not dramatically different from the observed scale of sampling variation (observed in this study) of an overall profile basal form
- Two groups of ever increasing post-horizontal ramus investment designs are clear, with the basal rami of *Chortoglyphus arcuatus* CH1, *Thyreophagus entomophagus* TH3, *Rhizoglyphus robini* R1, *Glycometrus hugheseae* G3 and *Dermatophagoides pteronyssinus* D3 being taller and sometimes more rounded than those of the distinct group *Acarus gracilis* A4, *Suidasia pontifica* S5, *Glycyphagus domesticus* G5, *Lepidoglyphus destructor* G6 and *Aleuroglyphus ovatus* AL2.

Note that the transects discovered generally go from ‘prickle-tip’ (proud of the data cloud) to ‘prickle-tip’ (proud of the data cloud), or ‘prickle tip’ (proud of the data cloud) to a member of the ’distinct group’ (the latter usually sharing a ‘slab-like’ basal form). Only the third candidate path (*Rhizoglyphus robini* R1$\rightarrow$*Tyrophagus putrescentiae* T13) and the fourth candidate path (*Chortoglyphus arcuatus* CH1$\rightarrow$ *Tyrolichus casei* T62) plunge in towards the centre of the data cloud. In other words, the various ‘prickliness’ is in somewhat different directions across the multi-dimensional data cloud.

**Fig. 21:**
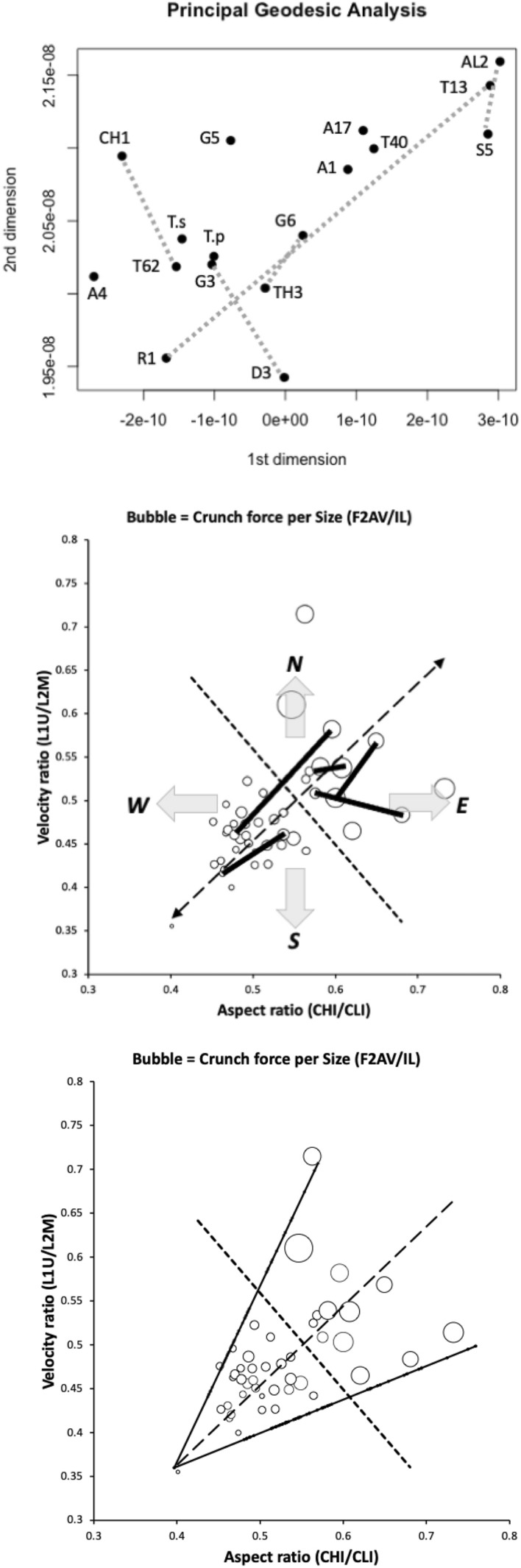
Interpretation of results. Upper: Display in two dimensions of principal geodesic analysis of $\Omega$ matrices over all taxa. Grey lines are linear approximation to projected geodesics (note drawn lengths not representative of true *lengeodesic* due to multidimensional ’star-like’ nature of data cloud. Middle. Geodesics overlain on Fig. 3 showing only the NW to SE second candidate transitional path ($TH3\leftrightarrow G6$) through moveable digit $\Omega$ design space is distinct from the general SW to NE geometric similarity. Amended from Bowman (2021) with permission, Creative Commons Attribution 4.0 International License http://creativecommons.org/licenses/by/4.0/). Lower. Symmetric triangular shaped envelope (solid lines) of astigmatan trophic design space from Bowman (2021) with permission, Creative Commons Attribution 4.0 International License http://creativecommons.org/licenses/by/4.0/

This is nicely confirmed in Fig. 21 where a flat two dimensional projected display of a principal geodesic analysis over the $\Omega$ matrices for all taxa shows no star-like or nested arrangement for the taxa nor for the candidate (projected) geodesics. The data cloud is not of a regular ‘globular’ shape, although those to its exterior usually share the rounded basal ramus design.

Indeed all of these design directions, bar one (*Thyreophagus entomophagus* $TH3\leftrightarrow$ *Lepidoglyphus destructor* *G*6) are aliased with the general geometric similarity of the chelicera. There is an important subtlety in the latter exceptional transformation in Fig. 16 between TH3 and G6 compared to the other candidate transitional paths for this upper group of larger mites (i.e., above the dashed line). This moveable digit profile shift correlates with an increasing chelal velocity ratio when the chelicera becomes more slender, elongate and pointed across the bird nest astigmatans, or conversely as the chelicera becomes more robust (and powerful) across these larger species there is a fall in chelal velocity ratio (all within a particular overall design type). Examining the triangular envelope of astigmatan design space (Fig. 21 Lower), one could say that in bird nest free-living mites: ‘There are few design ways to be small, weak and dainty but more ways to be trophically differentiated when large, powerful and robust’. This mirrors the conclusions about mesostigmatid miniaturisation in Bowman (2023b).

### Horizontal ramus features

The plesiomorphic form of the astigmatan moveable digit is usually assumed by acarologists to be edentate (approximating that of *Carpoglyphus lactis*, Bowman 2024b). Then, if one gullet formed behind the tip during evolution, then the mastication surface would become ‘claw-like”. Conversely if the mastication surface was raised the digit would become ’breasted’. Contrast *a* looks for between species evidence of these two evolutionary options of a single ‘long’ asperity (i.e., up or down overall).

Given that during evolution the astigmatan mastication surface might be ‘breasted’ (or conversely ‘anti-breasted’), then a gullet on the former or a tooth on the latter could form anywhere along its surface. This is tested for by contrasts $b1-b4$ and $c1-c7$ (at different scales). These look for three features (matching an up, down, up module or a down, up, down module). Logical reflection confirms that two such features are not possible. If three features exist, then further trophic evolution could have occurred within the ‘up’ regions or the ‘down’ regions. To investigate these further then one has the difficult issue of either very high density sampling of the surface (beyond that of this study) or a switch to evaluating the *observed* asperities themselves (in a follow-up study) as objects of a variable location. Being able to compare such across species raises the issue of homology, is this distal feature in species ‘A’ to be matched with that particular proximal feature or that particular distal feature in the other species ‘B’? Such is critical if one wishes to objectively use geometric morphometrics.

**Fig. 22:**
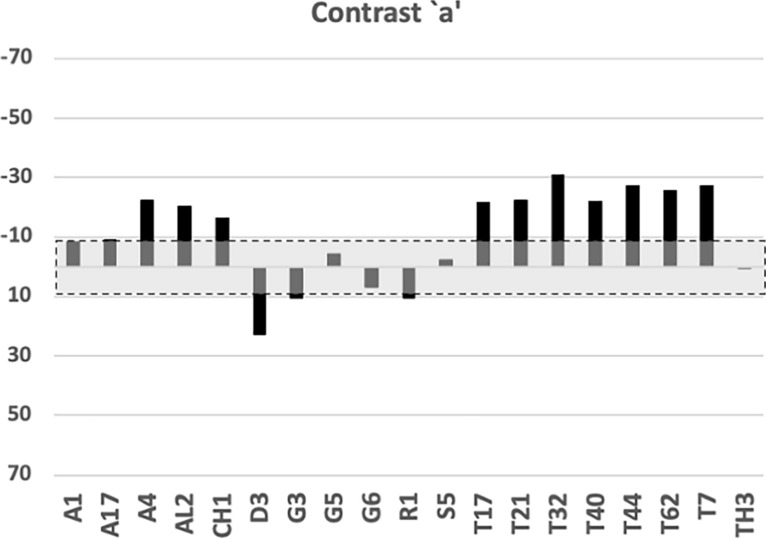
Horizontal ramus contrast *a*. Alphanumeric order of species. Grey zone with dashed outline is two sided $95\%$ confidence interval around zero showing non-significance for that contrast. The mastication surface in *Acarus gracilis* A4, *Aleuroglyphus ovatus* AL2, *Chortoglyphus arcuatus* CH1, *Tyrophagus putrescentiae* T13, *Tyrophagus palmarum* T17 & T32, *Tyrophagus longior* T40, *Tyrophagus similis* T21 & T44, and *Tyrolichus casei* T62 is clearly ’breasted’. That of *Dermatophagoides pteronyssinus* D3 is ’anti-breasted’. The tooth row of the remaining species is essentially level overall

**Fig. 23:**
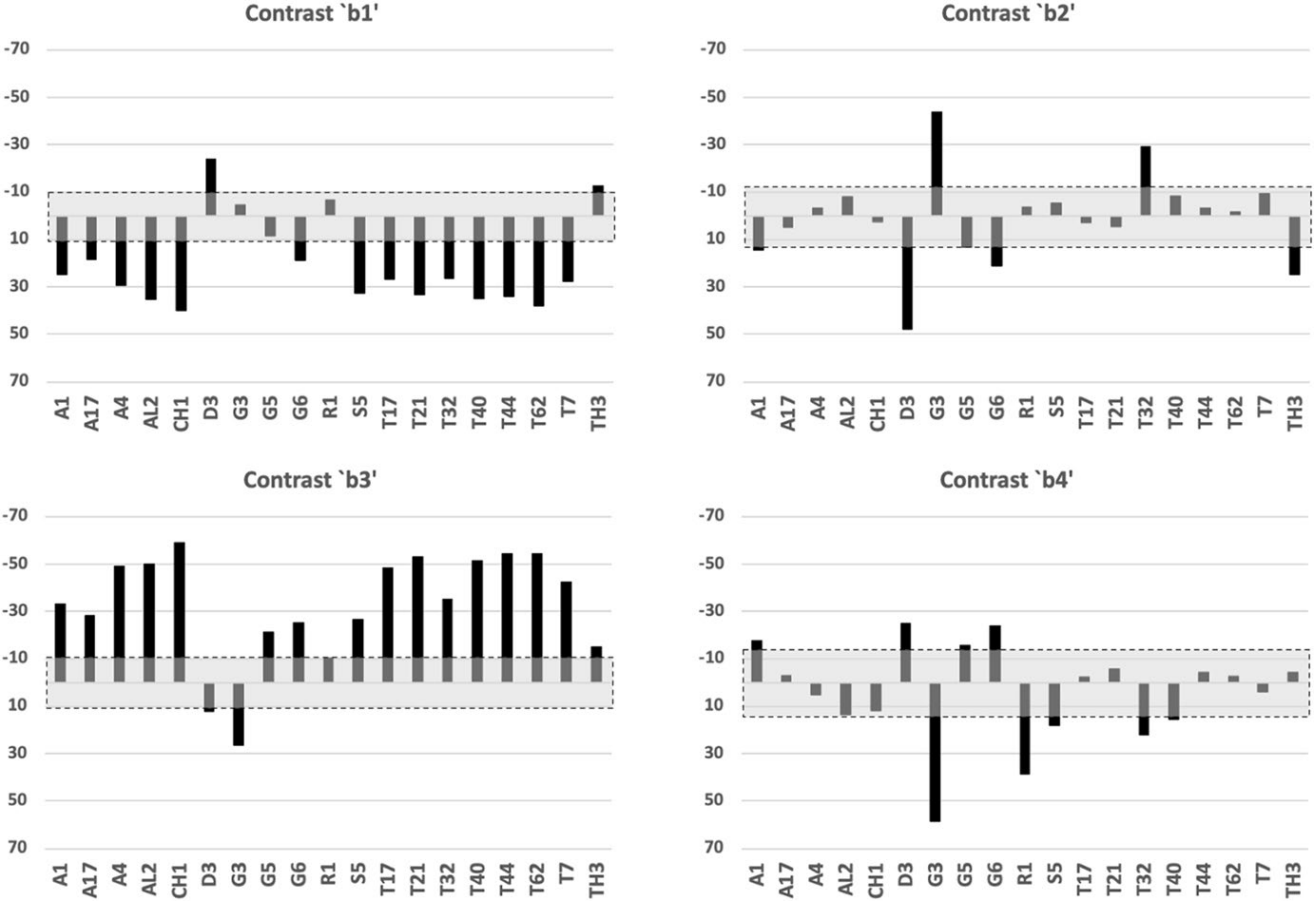
Horizontal ramus contrasts $b1-b4$. Alphanumeric order of species. Grey zone with dashed outline is two sided $95\%$ confidence interval around zero showing non-significance for that contrast. For contrast *b*1: The mastication surface in *Acarus immobilis* A1, *Acarus farris* A17, *Acarus gracilis* A4, *Aleuroglyphus ovatus* AL2, *Chortoglyphus arcuatus* CH1, *Lepidoglyphus destructor* G6, *Suidasia pontifica* S5, *Tyrophagus putrescentiae* T13, *tyrophagus palmarum* T17 & T32, *Tyrophagus longior* T40, *Tyrophagus similis* T21 & T44 and *Tyrolichus casei* T62 is clearly ’gulleted’ in the distal quarter of the mastication surface. That of *Dermatophagoides pteronyssinus* D3 is ’peaked’. The remaining four species are essentially level in this region. For contrast *b*2: The mastication surface here is clearly ’peaked’ in *Glycometrus hugheseae* G3, *Tyrophagus palmarum* T32 and clearly ’gulleted’ in *Dermatophagoides pteronyssinus* D3, *Lepidoglyphus destructor* G6 and *Thyreophagus entomophagus* TH3. However the majority of species are essentially level over this region. For contrast *b*3: the mastication surface is invariably peaked over this region. Only *Glycometrus hugheseae* G3 is strongly gulleted here. *Dermatophagoides pteronyssinus* D3, *Rhizoglyphus robini* R1 and probably *Thyreophagus entomophagus* TH3 are essentially level over this region. For contrast *b*4: *Dermatophagoides pteronyssinus* D3, *Lepidoglyphus destructor* G6 and perhaps *Acarus immobilis* A1 and *Tyrophagus putrescentiae* T13 are clearly ’peaked’ in this proximal quarter of the mastication surface. Three species, *Glycometrus hugheseae* G3, *Rhizoglyphus robini* R1, *Tyrophagus palmarum* T32 clearly and perhaps *Suidasia pontifica* S5 and *Tyrophagus longior* T40 are ’gulleted’ here. The remaining twelve species are essentially level over this region

**Fig. 24:**
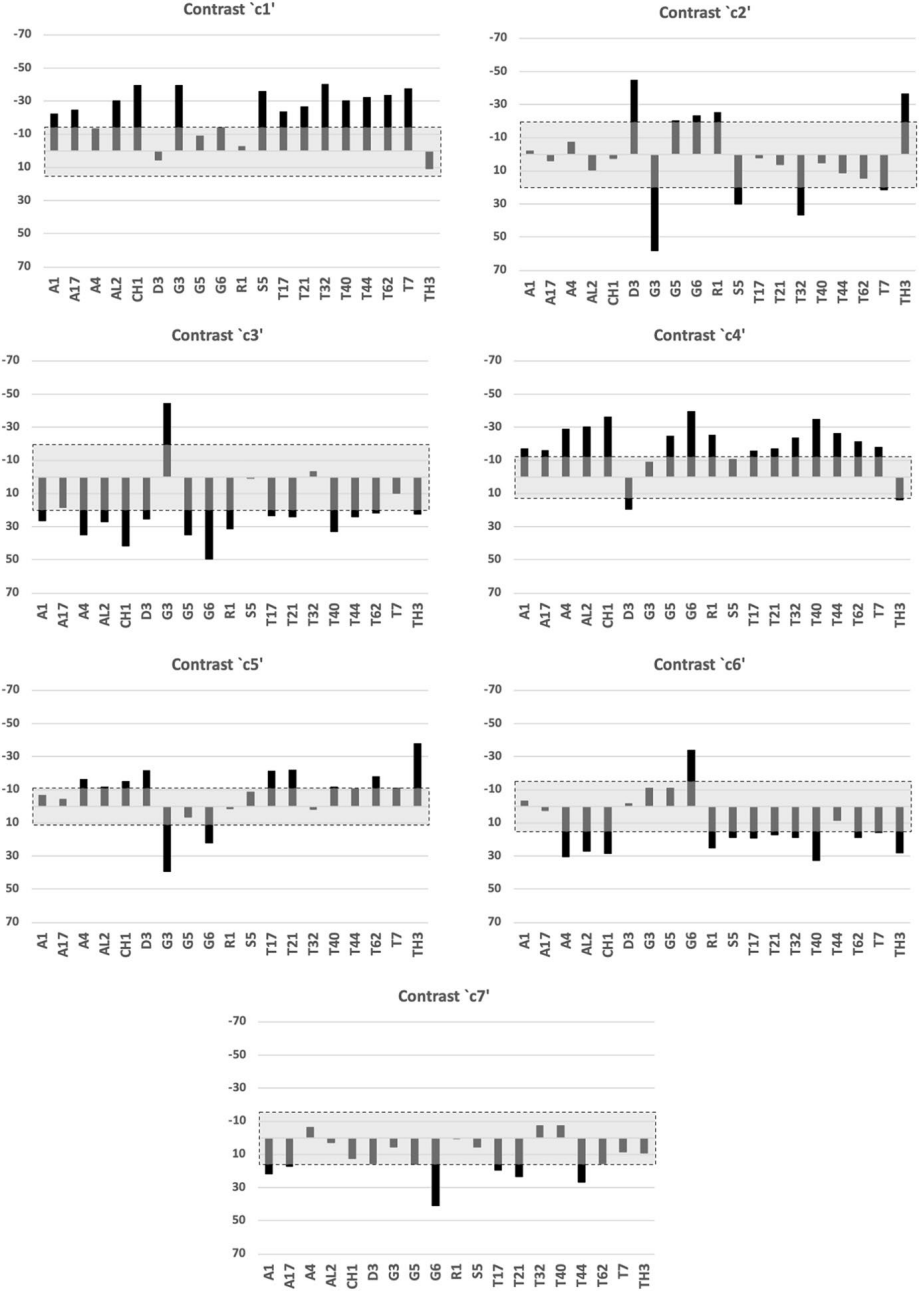
Horizontal ramus contrasts $c1-c7$. Alphanumeric order of species. Grey zone with dashed outline is two sided $95\%$ confidence interval around zero showing non-significance for that contrast. For contrast *c*1: All species are significantly ’peaked’ except *Acarus gracilis* A4, *Dermatophagoides pteronyssinus* D3, *Glycyphagus domesticus* G5, *Lepidoglyphus destructor* G6, *Rhizoglyphus robini* R1, *Thyreophagus entomophagus* TH3 which are essentially level distally. For contrast *c*2: *Dermatophagoides pteronyssinus* D3 and *Thyreophagus entomophagus* TH3 are clearly ’peaked’ just here and perhaps *Lepidoglyphus destructor* G6, *Rhizoglyphus robini* R1 too. The three taxa *Glycometrus hugheseae* G3, *Suidasia pontifica* S5 and *Tyrophagus palmarum* T32 appear to have a significant ’gullet’ here. For contrast *c*3: Only *Glycometrus hugheseae* G3 shows a significant ’peak’. At least *Acarus gracilis* A4, *Chortoglyphus arcuatus* CH1, *Glycyphagus domesticus* G5, *Lepidoglyphus destructor* G6, *Rhizoglyphus robini* R1 and *Tyrophagus longior* T40 show a significantly ’gulleted’ feature here. *Aleuroglyphus ovatus* AL2 may be gulleted too. The remaining species are barely gulleted or effectively level. For contrast *c*4: Only *Dermatophagoides pteronyssinus* D3 (and perhaps *Thyreophagus entomophagus* TH3) show a significant ’gullet’ at this location. Most of the species (bar the effectively level surfaced *Glycometrus hugheseae* G3 and *Suidasia pontifica* S5) are ’peaked’ to varying extent. For contrast *c*5: Only *Glycometrus hugheseae* G3 and *Lepidoglyphus destructor* G6 show a significant gullet hat this location. *Thyreophagus entomophagous* TH3 for sure is significantly peaked here. The remainder species have an effectively level or an equivocal ’peaked’ feature. For contrast *c*6: Only *Lepidoglyphus destructor* G6 is significantly ’peaked’ at this location. The majority of the species (bar significant ’gullet’ features in *Acarus gracilis* A4, *Aleuroglyphus ovatus* AL2, *Chortoglyphus arcuatus* CH1, *Rhizoglyphus robini* R1, *Tyrophagus longior* T40 and *Thyreophagus entomophagous* TH3) are effectively level or only possibly slightly depressed in this region. For contrast *c*7: *Acarus immobilis* A1, *Lepidoglyphus destructor* G6, *Tyrophagus putrescentiae* T13 and *Tyrophagus similis* T21 & T44 show a significant ’gulleted’ feature at this proximal location. The remaining species are effectively featureless (or only slightly depressed) here

**Fig. 25:**
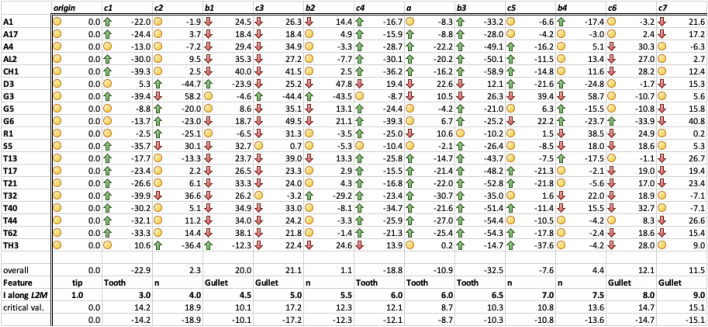
Summary of horizontal ramus contrasts ordered by centre of contrast along *L*2*M* axis indicating overall apparent asperities. Arrow up = profile raised (’peak’). Arrow down = profile sunken (’gullet’). Circle = not significant (two-sided $95\%$ confidence interval). Alphanumeric order of species. n = equivocal overall feature

The results of applying the contrasts to $\phi _{i}$ angles are shown individually in Figs. 22, 23 and 24 and summarised in Fig. 25. Blocks of commonalities between species are clear, defining where the apparent moveable digit asperities occur.

For overall ‘breasting’ (contrast *a*) of the mastication surface and for regional contrasts $b1-b3$ the two samples of *T.palmarum* T17 & T32, as well as the two samples of *T.similis* T21 & T44 are consistent. However, for the proximal contrast *b*4 the two samples of *T.palmarum* T32 & T17 disagree. The latter result suggests that the within species estimated variation (from the ANOVA) does not quite encompass the true between sample variation (perhaps due to the resultant positioning of the ascending ramus subtly changing within any mastication length variation) and so confidence intervals should be widened somewhat. This means that proximally the apparent significance of regional features for at least *Suidasia pontifica* S5 (with an apparent gullet) and *Acarus immobilis* A1 (with an apparent peak) should be discounted a bit. This inconsistency also arises for the local feature tested by contrast *c*2. Not only should the *Tyrophagus palmarum* T32 value be discounted a bit but also by increasing the variation, the apparent ‘peaks’ for *Lepidoglyphus destructor* G6 and *Rhizoglyphus robini* R1 at least should also be discounted a bit. Contrast *c*3 shows the same inconsistency for the apparent ‘gullets’ suggesting that the detection of apparent gullets in many species should be discounted a bit by somewhat widening the confidence interval again. Indeed these last two regions already show relatively higher confidence intervals than other similarly scaled contrasts suggesting a greater degree of plasticity ($\equiv$ horizontal ambiguity) of features within some species just here. Contrast *c*5 also has inconsistent scalar values for *T.palmarum* T17 & T32 (and to some extent for *T.similis* T21 & T44) pointing to a necessary widening of the confidence interval and the discounting a bit of the smaller ‘peaked’ results. Finally, contrast *c*7 shows inconsistent *T.palmarum* T7 & T32 results and the need for consequent discounting a bit of the smaller results that might at first appear to be outside of the confidence interval. Examining the location of actual asperities in a follow-up study is indicated.

What might these results above mean for the *pattern* of apparent asperities? One could propose from the summary row of ‘Features’ in Fig. 25 that a possible geometric morphometric approach for the mastication surface in the future could perhaps consider using the following locations as landmarks: moveable digit tip, first tooth posteriorly behind it, and last gullet before end of mastication surface plus the tooth anterior of it?

**Fig. 26:**
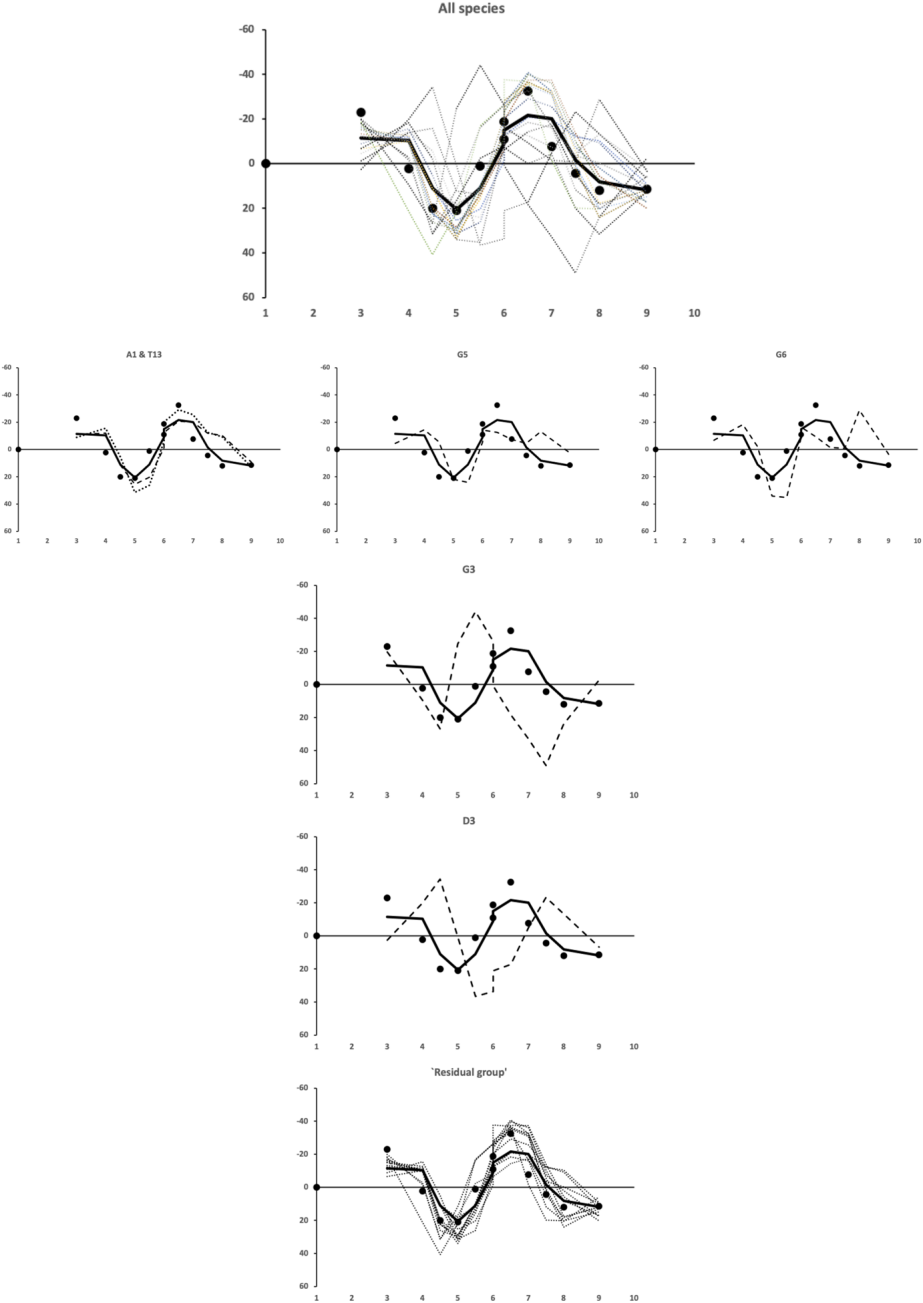
Smoothed mastication surface profiles. *x*-axis sampled location along *L*2*M* axis (1 = moveable digit tip). *y*-axis scalar from contrast centred at that location. Black dots and solid line average over species. Dashed lines individual species average profile. Up the page indicates a raised region (i.e., peaked), down the page indicates a depressed region (i.e., gulleted). Top row: All species. Second row: Species with lack of proximal gullet, peaked here instead (increasing left *Acarus immobilis* A1 & *Tyrophagus putrescentiae* T13 through *Glycyphagus domesticus* G5 to right *Lepidoglyphus destructor* G6). Third row: *Glycometrus hugheseae* G3 with proximal *L*2*M* axis expansion and distal compression resulting in exaggerated proximal gullet and heightened anteriorly shifted main peaked region behind ’notched’ first gullet. Fourth row: *Dermatophagoides pteronyssinus* D3 with distal *L*2*M* axis expansion and proximal compression resulting in posteriorly shifted main peaked region and diminished proximal gullet. Bottom row: Residual group without *Acarus immobilis* A1, *Dermatophagoides pteronyssinus* D3, *Glycometrus hugheseae* G3, *Glycyphagus domesticus* G5 and *Lepidoglyphus destructor* G6 showing common astigmatan mastication surface ’Bauplan’

Plotting the two-point moving average smoothed values from Fig. 25 gives Fig. 26.

Certain species (*Acarus immobilis* A1, *Dermatophagoides pteronyssinus* D3, *Glycometrus hugheseae* G3, *Glycyphagus domesticus* G5, *Lepidoglyphus destructor* G6) and *Tyrophagus putrescentiae* T13 have an individualised distinctly featured mastication surface. These species must feed differently or on different material in the bird nest habitat. However, the bulk of the species have a common *pattern* of apparent asperities on their mastication surface. Although, two species, *Rhizoglyphus robini* R1 and *Chortoglyphus arcuatus* CH1, have somewhat exaggerated features on this common ‘Bauplan’ (*plots not shown*) perhaps scaled for greater force.

## Discussion

For the most part, measurement errors appear small in this study and the mite specimens examined agree with those previously studied (Bowman 2021, 2023a, 2024a, 2024b). The new methods introduced herein appear robust and yield some crucial insights worth discussing. Parameters with a high inter-individual variation would offer the opportunity for individualised trophic niches in the community, i.e., the ability to minimise within species competition. Evidence for this phenotypic plasticity (and so possible polymorphic patches of groups of different mites in nests in the wild) is not strong at the profile level (although digit tip angle can be variable in some species Fig. 13).

As Woodroffe (1953) describes, ”...nest materials comprise organic matter of both animal and vegetable origin.”. At least for ptyctimous mites (Acari: Oribatida), nests of song-birds are special microhabitats, due to specific microclimatic conditions and accumulation of organic matter that engenders a different species composition than surrounding soils (Niedbała et al. 2023). This may be particularly so for sheltered (dry) nests which as Woodroffe 1953) says ”...decompose so slowly that they persist in their original condition for considerable periods and can consequently form a source of food for a more or less permanent population of those insects and mites that are able to thrive on dried organic materials.”. This may be the case even for the exposed twig nests of raptor species studied by Maciej Skorupski at Poznań University in Poland (albeit the fast drying would require xeric tolerant astigmatans). Conversely sun exposure with rain saturation rapidly reduces abandoned nests via bacterial and fungal decomposition to a fine ”...humus bound together by the coarser fibrous materials...” (Woodroffe 1953) and ”...a fauna similar to that of decaying vegetable matter in a wide variety of other situations.” (Woodroffe 1953). Astigmata are expected to be enriched and opportunities for morphological divergence over time to facilitate scavenging co-existence could be expected to be present in bird nests. Using the geodesic approach outlined herein, morphological reversion (aka vestigialisation) and independent spontaneous reappearance of features during any evolutionary change of course cannot be detected.

Of course, one could criticise the assumption used herein, that similar niche space inherently infers competition, as in practice it is impossible to accurately determine total niche space. So, just as in bat ecomorphology, there is always a need for detailed fieldwork to obtain a realistic and biologically meaningful view of a species’ diet (Swartz et al. 2003). One must also avoid over-simplified ecological categorisations (Birn-Jeffery et al. 2012). Not only should other aspects of morphology and proportions be taken into consideration, but of course even mites designed to specialise in efficiently processing specific foods (that are thus perhaps easier to access and exploit for them), must also have ‘fall-back options’ of feeding upon general foods (albeit with some difficulty). So compromises in trophic design and apparent paradoxes (as in cichlid fish Liem 1980) are to be expected.

### Why have a ‘slab-shaped’ basal ramus rather than a more rounded circular design?

This digit design question is exemplified by the *Acarus* spp. studied herein versus the glycyphagids studied. So this may represent a fundamental phylogenetic difference.

Consider the moveable digit to be like a sword. Experience by others (see George Turner, Part 2 at https://www.thearma.org/spotlight/GTA/motions_and_impacts2.htm) shows, as expected by elementary physics, that ”...heavy objects are harder to get moving than light objects, so heavy swords...” (or moveable digits) ”...are harder to accelerate...” (whether in a stabbing or a slashing direction) ”...than light ones.”. Therefore, the mass of the acarine moveable digit should always be focused in the basal ramus within the cheliceral base. Indeed, optimising the ‘pushing’ momentum of a tool like a saw on movement forward (aka cheliceral protrusion) requires (like George Turner, Part 2 at https://www.thearma.org/spotlight/GTA/motions_and_impacts2.htm says) a heavy handle as ”...once in motion, heavy objects are likewise harder to stop...” (by say flesh or foodstuff resistance). Accordingly (as can be verified in any hardware iron-mongery store) saw handles are thus usually slab-shaped.

Openly available material (see George Turner, Part 1 at https://www.thearma.org/spotlight/GTA/motions_and_impacts.htm)) is useful to explain matters here. A heavy pommel on a sword can increase stabbing momentum and makes it feel lighter and more manoeuvrable (https://www.reliks.com/functional-swords/european-swords/pommel/). However, a pommel on a sword optimises rotational behaviour rather than the widely held view that it is for balance (see George Turner, Part 3 at https://www.thearma.org/spotlight/GTA/motions_and_impacts3.htm). Optimising the moveable digit moment of inertia for this rotation around the condyle points to a generally circular basal ramus (Bowman 2024a) as best. The high $\alpha$ value *Glycometrus hugheseae* G3, *Glycyphagus domesticus* G5 and *Rhizoglyphus robini* R1 (Fig. 12 are matched to a rounder basal ramus (Fig. 6 suggesting they have a (rotational) crushing action moveable digit rather than a push and pull cutting or stabbing action.

Experience by others (see George Turner, Part 1 at https://www.thearma.org/spotlight/GTA/motions_and_impacts.htm) shows that one cannot have the effective pivot point of a weapon (like a striking action sword or therefore the impact of a rotating moveable digit) ”...very close to a weapon’s centre of mass, since it would mean that the corresponding impact point would move past the end of the weapon.” (and cause ‘hand shock’ on impact). Therefore, even if there were no condyle and the acarine digit was ‘free-floating’ in a moving cheliceral base, as George Turner, Part 1 at https://www.thearma.org/spotlight/GTA/motions_and_impacts.htm says ”...since an impact can‘t actually occur past the end of a weapon, there can‘t be an impact that produces a pivot point that is very close to the weapon’s balance point.”. This drives the need for a relative diminution of basal ramus mass posterior of the acarine condyle when piercing and slicing (like avoiding the design of a poorly swinging sword that has been balanced too far back). This may be also the case in the cutting style kill action of mesostigmatids. Clearly tapering the acarine moveable digit distally aggravates this issue.

The more the horizontal ramus is strengthened (so resisting induced forces on occlusion, Bowman 2024b) or the more the chela becomes ‘stubbier’ ($\Rightarrow$ higher *VR*) - the less any basal ramus asymmetry is needed (as the centre of mass moves). This may explain the tip angle results. Alternatively a well developed ascending ramus could be the compensatory factor. The relevance of the shape of the ascending ramus is discussed below.

### What about digit patterns and friction?

Gripping needs friction. Bowman (2024a) discusses friction and astigmatan digit designs. Pamfilie et al. (2023) showed in lizards that various aspects of claw shape have an influence on frictional interactions. However, Pamfilie et al. (2023) found that this was ”....only on substrates for which...” the lizard claws’ ”...asperities...” were of a size to produce a ”...mechanical interlocking with the claw”. In other words it was substrate context specific. Pamfilie et al. (2023) goes onto say ”On such substrates, the diameter of the claw’s tip...” was ”...the most important predictor of friction..”. Pamfilie et al. (2023) claims that ”...narrower claw tips induced greater frictional interactions than wider ones...” did. Pamfilie et al. (2023) also found that ”...claw curvature, length, and depth influenced friction, but that these relationships...” again depended upon ”...the substrate’s surface roughness.”.

For the bird nest astigmatans reviewed, moveable digit tip diameter is indicated by the tip angle, as the tip diameter ($\delta$ units back from its point centrally) is by simple trigonomertry $=2.\delta .tan(\frac{tip\ angle}{2})$. So the ordering of this set of bird nest astigmatan species by moveable digit tip diameter does not change from that arrangement (shown in Fig. 13) within the angle-based ‘digit sharpness’ space. Only the species relative proximity to each other is changed non-linearly (i.e., any clumping at high angle values are stretched out). High angles should induce stronger friction against foodstuffs (all other matters being equal).

**Fig. 27:**
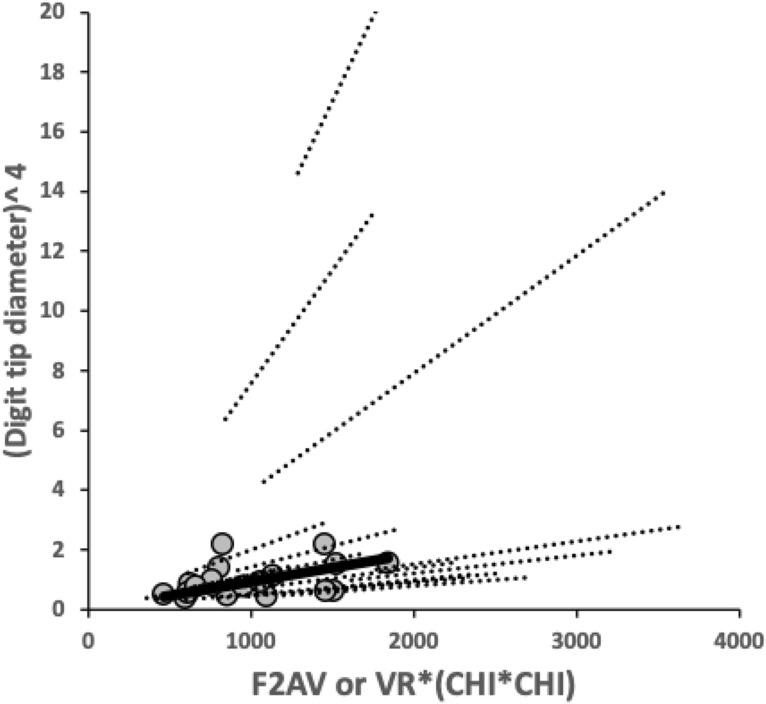
Plot of moveable digit $(tip\ diameter)^{4}$ versus adductive force at the digit tip (*F*2*AV* from Bowman 2021 for the species mean, or $VR*(CHI^{2}$) for each individual within a taxon) for the sample used herein. Solid line and grey dots = mean data. Dashed lines = linear regression fits through zero for individual species separately. The three upper grey dots are for *Acarus gracilis* A4, *Glycyphagus domesticus* G5 and *Lepidoglyphus destructor* G6. The upper within taxon regressions are for the digit tips of *Lepidoglyphus destructor* G6, *Suidasia pontifica* S5 and *Glycometrus hugheseae* G3 in increasing rate order

Of course digit tip diameter should be greater for those moveable digit tips undergoing higher (*F*2) loads (c.f. the basal filament being thicker in the fibre model of digit strengthening in Fig. 21 of Bowman 2024b). Loads are resisted (transversely) by the squared cross-sectional area of an object. Now the flexural rigidity ($=E.\mathbb {I}[X]$) near the tip (for similar Young’s moduli over taxa, see Bowman 2024a) can be approximated as $\propto \frac{1}{(tip\ diameter)^{4}}$ (at a standard $\delta =1$). Or, under the null hypothesis of no extra evolutionary changes, one expects approximately that $F2_{tip}\propto (tip\ diameter)^{4}$. There is mild evidence that this is true for this set of bird nest astigmatan species (overall across species $R^{2}=0.7936$) and for each taxon (Fig. 27). *Acarus gracilis* A4, *Glycyphagus domesticus* G5 and *Lepidoglyphus destructor* G6 have more than expected strengthened digit tips compared to other taxa. However, it is the digit tips of *Lepidoglyphus destructor* G6, *Suidasia pontifica* S5 and *Glycometrus hugheseae* G3 that become robustified at a noticeably higher rate than the typical astigmatan rate as adductive loads increase.

Now ‘saw depth’ $W\approx \frac{depth\ of\ likely\ dentition\ base\ plate}{1-\frac{1}{b}}$ where $\frac{1}{b}$ is the *common ratio* (Bowman 2024b). By setting $tip\ diameter=depth\ of\ likely\ dentition\ base\ plate$ (i.e., assuming a non-curved up moveable digit tip) one can estimate the parameter *b* (at a standard $\delta =1$). This parameter represents the overall curvedness of the venter of the moveable digit. For this set of bird nest astigmatan species, each species does not differ widely from the overall between-taxa relationship of $\hat{b}=1.098$ (*plot not shown*). Moveable digit ventral surface intrinsic curvature appears common across species.

Furthermore, recall from Bowman (2024a), that like in insects (Petie and Muller 2007), in comparison to straight designs, curved profiles can encourage food material to be driven back towards ‘jaw’ pivot points (and not away from the animal on occlusive ‘strike’). This obviates the need for friction playing a major part in holding material as the digit and food stuff surfaces move against each other.

### What might the steepness of the ascending ramus indicate?

Classic vertebrate ecomorphological studies of jaws (e.g., Morales-García et al. 2021) have seemingly not explicitly considered the slope of the ascending ramus, restricting themselves to conclusions like tall ascending rami are found on herbivores’ shorter jaws, insectivores have short ascending rami and longer jaws etc. However, the usual consideration of elastic percussion (‘thumping’) versus cutting by any moving surface would pertain to the form of the ascending ramus when mite chelicerae are protruded into material (if food can indeed reach this surface). Case-by-case consideration related to the context of actually how a mite feeds is needed (e.g., skimming in *Carpoglyphus lactis*). For sure, *Acarus farris* A17 has a shallow ascending ramus (Fig. 6 for example), while the glycyphagids have a much steeper form. Explicit measurement and modelling is needed with special contrasts (see Materials and Methods) allowing for the differences in $x_{i_{e}}$ between species in follow-up work.

One possibility for this is that as Bowman (2024b) shows, moveable digit depth is related to resisting bending stresses on occlusion (much as in primate mandibles Hylander 1979), if evolution favoured an essentially horizontal ventral surface to the moveable digit so as to allow it run along. a level ‘floor’, then one could strengthen a slicing action digit (proximally to the condyle) by increasing morphological investment over the ascending ramus steepening it in compensation. A follow-up finite-element modelling study (like Marcé-Nogué 2020) is indicated.

**Fig. 28:**
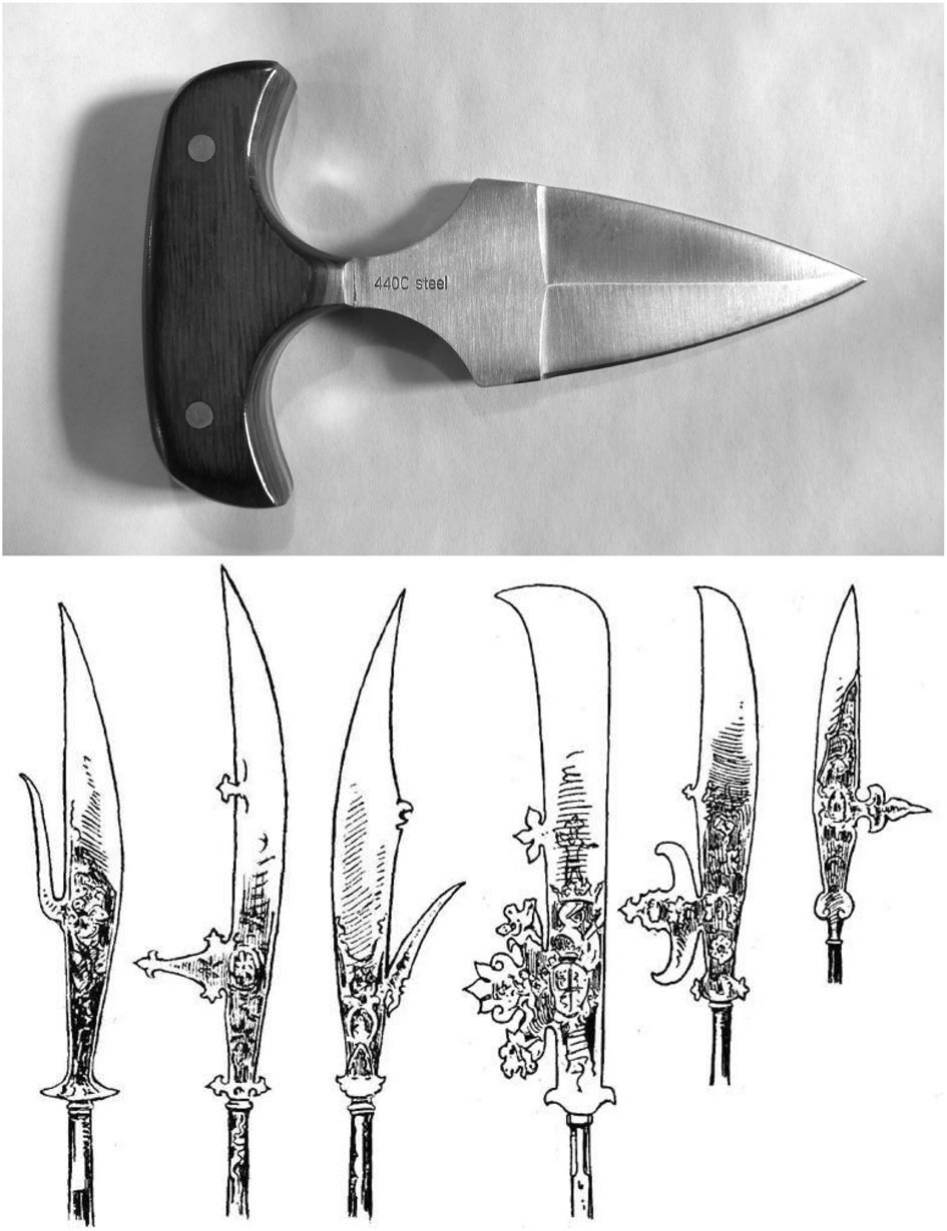
Analogues of the moveable digit’s ascending ramus. Upper. Push dagger (George Chernilevsky 25 April 2009 public domain image ex https://en.wikipedia.org/wiki/Push_dagger. User’s fist punches weapon forward. Note t-shaped handle matching a double-sided (i.e., reflected around moveable digit *L*2*M* axis) steep ascending ramus. Lower. Various glaives amended from Boeheim (1890) - public domain image from https://en.wikipedia.org/wiki/Glaive

Indeed, a steep ascending ramus (if accompanied by a sharp blade and a pointed tip) matches that design of (the upper half of) a ranseur (https://en.wikipedia.org/wiki/Ranseur) or the handle of a ‘push-dagger’ (Fig. 28 Upper). The profile shape of any lateral structures (in and around the ascending ramus of the moveable digit) will matter in astigmatans (Fig. 28 Lower).

It may be the same design as the function of the lateral adornments on glaives where forward leaning bladed structures would be able to slice into material trapped in the blade’s posterior notch as the pole-arm was thrust forward. Trapped material can then be twisted loose. Lateral protrusions on halberds (https://en.wikipedia.org/wiki/Halberd) and partisans (https://en.wikipedia.org/wiki/Partisan_(weapon)) were similarly used to hook opponents in combat.

**Fig. 29:**
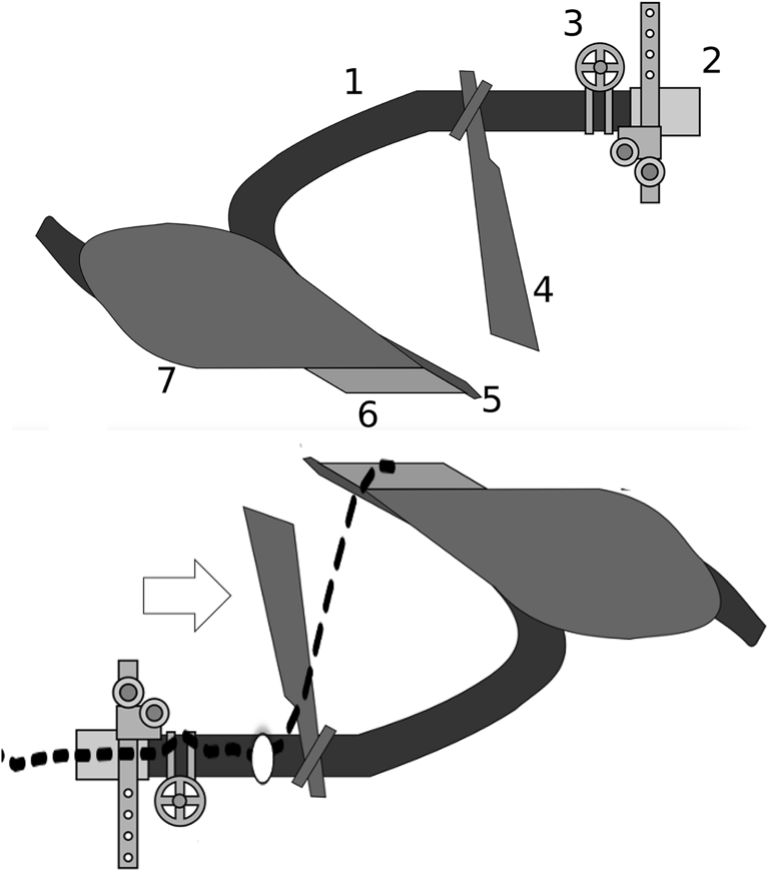
Upper. Diagram of a plough amended from public domain image https://en.wikipedia.org/wiki/Plough under 1 = beam. 2 = hitch. 3 = vertical regulator. 4 = coulter (knife coulter pictured, but disk coulter is common). 5 = chisel (foreshare). 6 = share (mainshare). 7 = mouldboard. Note commonality of $5\rightarrow 1$ topology with mesostigmatid design in Fig. 32. Lower. Inverted plough with average moveable digit profile of *Carpoglyphus lactis* from Bowman (2024b) superimposed as a dashed line (white dot = condyle). Open large arrow = flow of food material towards ascending ramus

Topologically the ascending ramus of digit is also like a ploughshare (Fig. 29) on a long pole or beam (so approximating a kama-yari - see https://en.wikipedia.org/wiki/Kama-yari).

The profile of a ploughshare or indeed just the knife-like coulter in advance of it (https://en.wikipedia.org/wiki/Coulter_(agriculture)) is designed to allow material to be sliced as it is drawn through. So a shallow angle to the ascending ramus (e.g., *Acarus farris* A17) will effectively undercut material on cheliceral protrusion with an open chela. Then depending upon the detailed left-right 3-D geometry, either turn the foodstuff material so as to spiral it in one lateral direction (like a plough) or force it equally sidewards (like a wedge) expanding the resultant groove in the food material. However, a vertical ascending ramus if narrow would simply slice food material without necessarily lifting or deflecting it (like a ‘digging stick’ or a hoe blade does). A blunt coulter (or ascending ramus) would simply ‘thump’ material which could bounce off of it. Indeed a blunt ploughshare blade will suffer increased traction resistance (through soil, Nuriev et al. 2022). This mechanical issue may therefore be important in nematode attack and tissue processing by astigmatan moveable digits. An SEM follow-up study is needed to investigate precise geometries in and around the posterior end of the mastication surface more.

### Which class of lever system matches which astigmatan species?

**Fig. 30:**
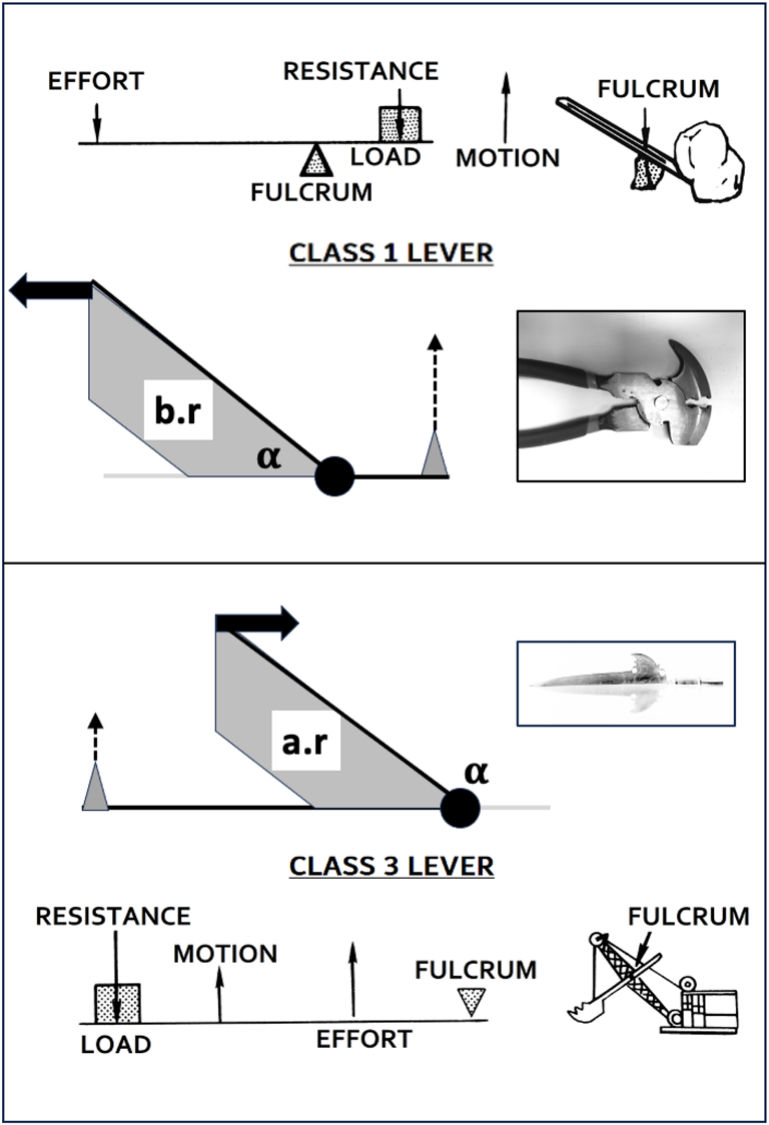
Two effective classes of lever can be found in mite moveable digits. The Class 1 lever is typified by ’see-saw’ and crushing pliers - Upper subfigure. The Class 3 lever is typified by a bucket excavation-digger and stabbing ’Officersbardisan’, public domain image of LRK 13054 from 1620s Göran Schmidt, Livrustkammaren (The Royal Armoury) - Lower subfigure. b.r = basal ramus in grey shading. a.r. = ascending ramus in grey shading. Black dot = articulating condyle. Large black arrow = adductive force *F*1 on cheliceral tendon. $\alpha$ = digit angle (see Akimov and Gaichenko 1976). Small grey triangle = digit tip at end of *L*2*M* output lever arm with dotted resultant tangential output force *F*2. Schemas in part extracted from public domain image by Pearson Scott Foresman (see https://en.wikipedia.org/wiki/Lever)

The detail of the overall acarine lever system for each taxon is important. Recall, from simple physics, that levers with high mechanical advantage (here = velocity ratio) can move large loads with a relatively small amount of effort (*F*1). Fig. 30 shows that two possibilities depending upon the $\alpha$ design angle (Fig. 12) are pertinent. Class 1 levers (https://en.wikipedia.org/wiki/Lever) includes see-saws and boat oars (and the raising of a human head by the neck muscles in order to head a football). Class 3 levers (https://en.wikipedia.org/wiki/Lever) include shovels and the raising of the human fore-arm plus hand around the elbow joint by the contraction of the biceps muscle, they can be relatively inefficient.

As energy must be conserved, in a frictionless system: $Work\ in=Work\ out$ or $F2*[dist.\, moveable\, digit\, tip\, moves]=F1*[dist.\, adductive\, tendon\, junction\, moves] \Rightarrow VR \equiv \frac{distance\, adductive\, tendon\, junction\, moves}{distance\, moveable\, digit\, tip\, moves}$.

**Fig. 31:**
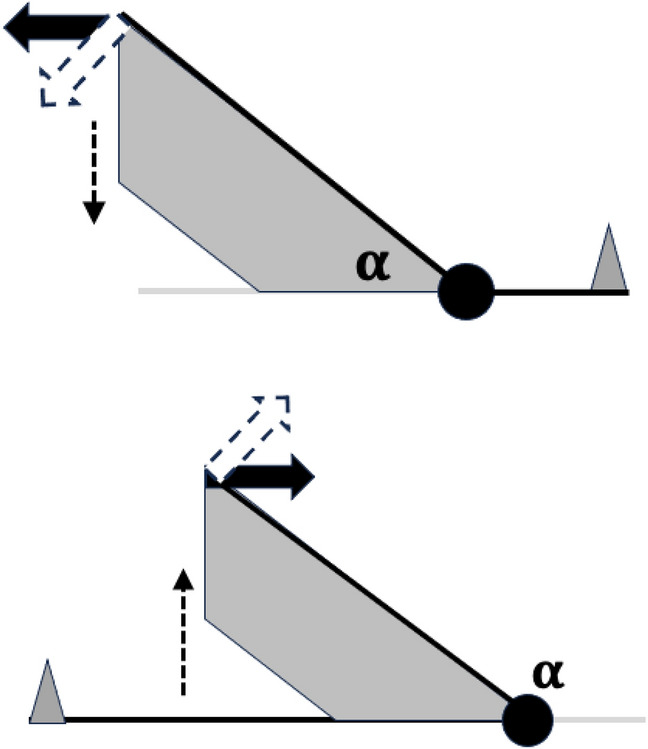
Chelal levers act differently according to the $\alpha$ design parameter angle (see Fig. 30). Upper Class 1 lever where adductive force (black arrow) is resolved to open-arrow movement and downward compressive stress (dashed arrow) on basal ramus. *L*1*U* here suffers possible extension. Lower Class 3 lever where adductive force (black arrow) is resolved to a force orthogonal to *L*1*U* generating the open-arrow movement and upward tensive stress (dashed arrow) on ascending ramus. *L*1*U* here suffers possible compression

Given an adductive force (*F*1) on the closing tendon, two scenarios arise (Fig. 31). In both cases the velocity ratio (*VR*) around the articulating condyle remains $=\frac{L1U}{L2M}$ and the $rotational\ torque\ in = pr(F1).L1U= rotational\ torque\ out = pr(F2).L2M$ where *pr*(...) means ‘perpendicular to the lever arm resolved’. Note therefore that as the cheliceral muscle orientation is on essentially a fixed alignment (within the basal shaft), just like pedalling a bicycle the rotational torque varies as the digit moves. In that way power delivered to the moveable digit tip varies according to relative orientations (as $power=torque*rpm$ where *rpm* is the effective revolutions around the condyle per time period). So torque is sacrificed for speed i.e., low *VR* indicates a design for rapid chelal occlusion, high *VR* a design for slow powerful crushing.

Moreover, the Class 3 lever has the opportunity for finer control (i.e., precision of motion) and greater speed in the load (https://en.wikipedia.org/wiki/Lever).

**Fig. 32:**
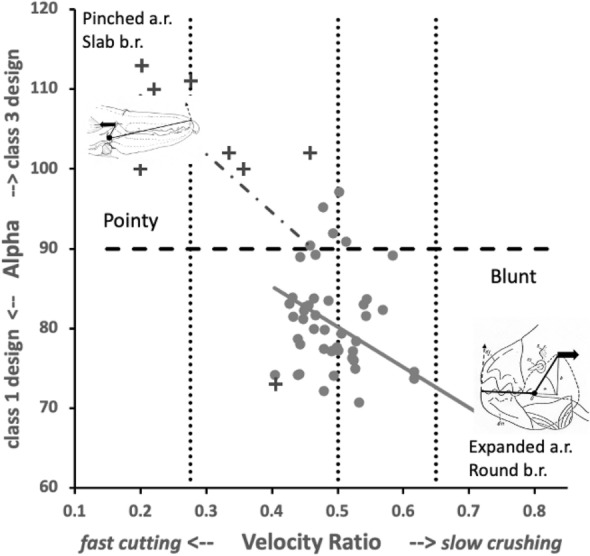
Data from Bowman (2020) and Bowman (2021) plotted by $\alpha$ design parameter angle. Cutting kill style mesostigmatids (crosses) are typified by ’pointy’ chelae with a ’pinched’ ascending ramus and a ’slab’ shaped (i.e., posteriorly abbreviated) basal ramus. Crushing feeding style astigmatans (grey circles) are typified by blunt chelae with an expanded ascending ramus and a more symmetrical sub-circular basal ramus. Note similar within group linear regression line slopes ($\approx -60$). The upper group of astigmatans includes *Glycometrus hugheseae* G3, *Glycyphagus domesticus* G5, *Lepidoglyphus destructor* G6 and *Rhizoglyphus robini* R1. Subfigures reproduced and amended with permission, Creative Commons Attribution 4.0 International License http://creativecommons.org/licenses/by/4.0/)

The fine detail of this of course depends upon the exact geometry of the mite’s occlusive musculature when the digit is open (as the subtended angles of resolved forces will change as the digit swings closed). However plotting the observed astigmatan data highlights the main determinants of digit design. Mite chelae are mechanically coherent for their intended function.(Fig. 32). Indeed the analysis of variance of the $\alpha$ results herein (Fig. 12) confirm that most bird nest astigmatans carry ‘crushing’ class 1 design chelae, only *Glycometrus hugheseae* G3, *Glycyphagus domesticus* G5, *Lepidoglyphus destructor* G6 and *Rhizoglyphus robini* R1 are designed as ‘cutting’ class 3.

**Fig. 33:**
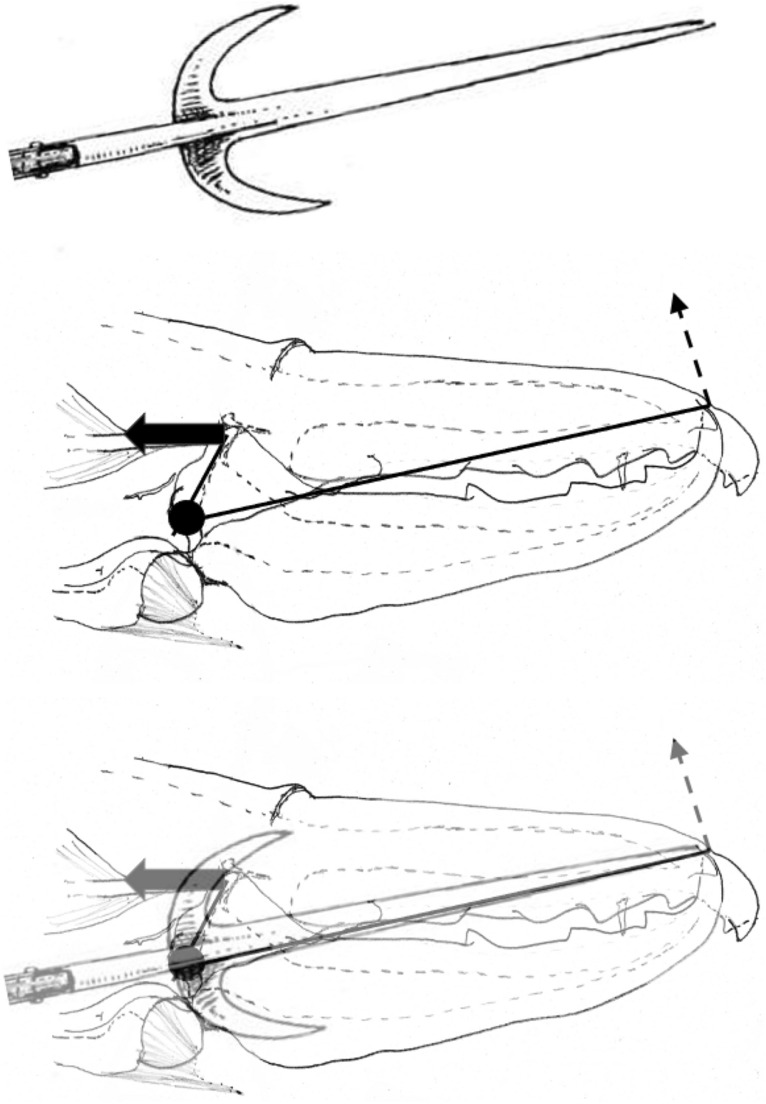
Cutting kill style chelae act like ranseurs (see https://en.wikipedia.org/wiki/Ranseur). Upper, a typical Ranseur, amended public domain image within the anthology including Boeheim (1890). Middle, chela from *Parasitus lunaris* DN ex Bowman (2020) with permission, Creative Commons Attribution 4.0 International License http://creativecommons.org/licenses/by/4.0/). Lower, semi-transparent overlay of both images showing match of lateral cutting projections to the geometry of the ascending ramus and its adductive tendon junction

The coulter analogy can be validated by a simple visual overlay (Figs.29 and 33). Indeed the ploughshare tip (5) and the arc around to the beam (1) in the upper sub-figure of Fig. 29 nicely matches the reflexed geometry of mesostigmatids shown in the top left of Fig. 32.

**Fig. 34:**
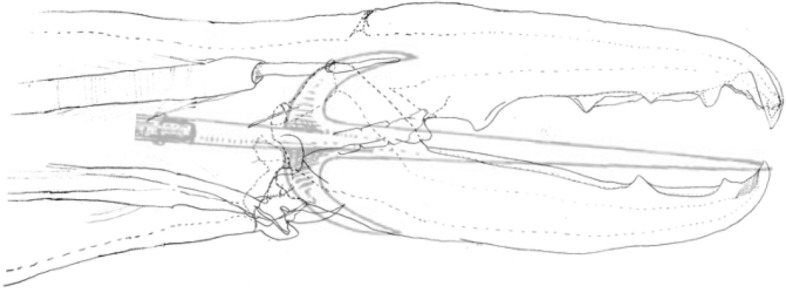
Both tangs of an overlain Ranseur match the geometry of effecting any chelal movement in *Veigaia nemorensis*. Chela from Bowman (2020) with permission, Creative Commons Attribution 4.0 International License http://creativecommons.org/licenses/by/4.0/). Ranseur from Fig. 33. Ascending ramus when chela is open will act like a coulter (see Fig. 29)

Indeed, in *Veigaia nemorensis* such an overlay shows that the chelal opening tendon junctions coincides similarly with the other tang of the ranseur (Fig. 34) supporting the view that such elongate chelae (with input moment arms $L1U \approx {\L } 1L$) could be as speedily opened as they are closed simply in terms of relative abductor to adductor muscle volumes. An action like whirling slashing knives in close-combat fighting in Kung Fu movies comes to mind.

### Future work

A simple start would be for acarologists to map moveable digit (longitudinal) dorsal half-profiles (see *Carpoglyphus lactis* in Bowman 2024a for an example) to already proposed weapon classification schemes (e.g., Dean 1916 for pole-arms - see https://en.wikipedia.org/wiki/Polearm) which may help explain the step-wise evolution of their function in mites.

In this study, digit tip adaptations are investigated. Bowman (2024b) already points to the possible decoupling of distal elongation from the differentiation of the more proximal dentate parts of the moveable digit profile. In vertebrates, specific morphogenetic processes are involved in digit tip development (Casanova and Sanz-Ezquerro 2007). Although the latter is involving bone rather than chitin, it would be very interesting to see if there are such similar mechanisms at play in mite embryology.

A more complicated approach could be to consider the complete moveable digit profile around its whole perimeter as a contour and deploy summary methods such as Fu et al. (1997) on each specimen before comparative analysis. However, various actions can be done within this framework of linear operators described in the Materials and Methods section.

For example:

- Between species, one could select which contrast maximises the variance between the set of species’ mean (over individuals) scalar values versus the overall mean using ANOVA
- Then for selected discriminatory features, one could test if a particular species is significantly different from another using an appropriate confidence interval
- Then, given that under the evolutionary null, the horizontal ramus should be ‘featureless’, one could estimate the most plesiomorphic digit as that with the least apomorphic differentiated modules of any size

Or

- One could pose over-lapping features i.e., the situation where modules of different sizes co-occur in some way, although care is necessary as the contrasts above are not necessarily orthogonal (so residual $\phi _{i}$ values given one contrast may be needed to isolate the specific extra contribution that one contrast gives over another if of interest)
- Or one could even carry out an overall TRANSECT analysis of a composite feature of interest, including multidimensional scaling, principal geodesic analysis and clustering (see Explanatory Appendix).

Many extensions are possible in future work

The ascending ramus and basal ramus is intentionally ignored in the contrasts used herein as neither trajectory is expected to be locally ‘toothy’, although for the last eight $\phi _{i}$ values to the condyle perhaps other more complicated contrasts could be posed which ignore certain locations e.g.,

$$\left( \begin{array}{ccccccccc} 1,1,0,-2,-2, 0,1,1 \end{array} \right)$$

scaled by $\frac{1}{\sqrt{12}}$ which could be used to indicate relative ‘breasting’ of the posterior digit shape inside the cheliceral shaft. Or perhaps plotting the scalar arising from using

$$\left( \begin{array}{ccccccccc} 1,1,0,0,0,0,0,0 \end{array} \right)$$

on the last eight $\phi _{i}$ values versus those using

$$\left( \begin{array}{ccccccccc} 0,0,0,0,0, 0,1,1 \end{array} \right)$$

both scaled by $\frac{1}{\sqrt{12}}$ for individuals and species could be useful? Here the first two coefficients are smoothing or averaging the ascending ramus slope, the last two smoothing or averaging the slope around the adductive tendon attachment. These ought to be $\approx 90^{\circ }$ different all other matters being equal, and could be tested in future work.

This study is the first laboratory validation of the analytical method (i.e., a TRL4 test, https://ec.europa.eu/research/participants/data/ref/h2020/wp/2014_2015/annexes/h2020-wp1415-annex-g-trl_en.pdf). What is needed now are detailed samples from bird’s nests in the field for a TRL5 validation of the analytical approach. In particular if a transition through the pest species is still supported. This offers the potential of detecting new possible pest species.

In particular, multiple isolates of different nidicolous species are needed from different origins in order to confirm if the apparent plasticity (Bowman 2024b) and high adaptive potential (Michalczyk-Wetula et al. 2021) of *T.putrescentiae* occurs in other astigmatans. For sure geographic-based genetic diversity, possibly due to climatic causes, is known in *D.farinae* (see Tao et al. 2024). However, as astigmatans have a high dispersal potential (via phoresy Seeman and Walter 2023), one would expect such arthropods to emigrate and immigrate without necessarily the need to become better adapted to changing local environmental conditions genetically (see Ponge 2020).

Indeed, the methods herein could be used to model moveable digit profile $\Omega$ matrices over ‘spatially sampled‘ geographic data, to assemble weighted interpolates and summaries (Fletcher and Joshi 2007) as well as Riemannian distance-based clustering of various types. Dealing with time-dependencies between $\Omega$ matrices (such as is needed to track evolutionary changes temporally) is outlined in Ben-David and Marks (2014). The methods could be extended to deal with relating morphological adaptations (described by Kerschbaumer and Pfignstl 2021) to environmental predictability as posited by Karasawa and Hijii (2004) for oribatid claws to see if this occurs for astigmatan chelae.

For sure, the species segmentation discovered in this laboratory-based set of individuals needs to be replicated by wild-collected specimens. Detailed fieldwork on actual diet could convert these morphological functional groups into guilds (Denzinger and Schnitzler 2013) and resolve multiple distinct morphologies (as in bats, Hedrick and Dumont 2018) for the same apparent niche. Niche partitioning by character displacement when in co-existent competition within a habitat is expected (much as the ecomorphological correlates of anole lizard claw shape changes, e.g., Yuan et al. 2020). Can this be detected in mites from wild nests with different complements of astigmatans in?

## Conclusion

In his general free-living astigmatan review, Bowman (2021) already trophically categorised the studied bird nest habitat species as follows

- Surface omnivores: R1, T62, T40, G6
- Surface fragmentary feeders: A1, TH3, T17, T21
- Interstitial omnivores: AL2, CH1, G3, G5, and
- Interstitial fragmentary feeders:A17, A4, T32, T13, T44, D3, S5.

Some extra characteristic must allow coexistence amongst each group.

The surface omnivores each sit in different design classifications (his Table 11 page 354) and should not trophically compete with each other.

*Thyreophagus entomophagus* TH3 has a unique surface fragmentary feeding design herein and can avoid trophic competition. However for the remaining surface fragmentary feeders, *Acarus immobilis* A1, *Tyrophagus palmarum* T17 and *Tyrophagus similis* T21, all sit in the same design class. The kerf, *thick*, tip and distal digit angle do not separate these from each other either. This needs explaining as to how coexistence is possible in the bird nest habitat in follow-up work.

All four interstitial omnivores (*Aleuroglyphus arcuatus* AL2, *Chortoglyphus arcuatus* CH1, *Glycometrus hugheseae* G3 and *Glycyphagus domesticus* G5) present in the bird nest habitat sit together in a single particular design class also apparently without direct trophic competition which also needs explanation (in followup work). There is some partial separation out from each other in this group with respect to kerf and characteristic digit angles (Fig. 10).

Amongst the interstitial fragmentary feeders, previously *Acarus farris* A17 was seen as unique trophically (Bowman 2021). *Dermatophagoides pteronyssinus* D3 and *Suidasia pontifica* S5 were also separate distinct trophic designs. Yet *Acarus gracilis* A4 and *Tyrophagus putrescentiae* T13 were classified together in one specific class, and the ‘basal forms’ *Tyrophagus palmarum* T32 with *Tyrophagus similis* T44 together in another. Although *Acarus gracilis* A4 and T13 (*Tyrophagus putrescentiae* sit in different whole profile clusters (Fig. 20), exactly how can the constituents of these latter groups co-exist in bird nests? There are still conundra that need solving.

So, whole moveable digit dorsal profiles and horizontal ramus (mastication surface) features are informative but still do not fully explain the ability of all free-living bird nest astigmatans to coexist trophically. More detailed investigation of ascending ramus shape and observed discrete dentition is needed.

## Figures and Tables

**Fig. 35:**
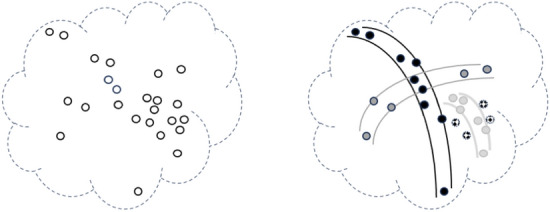
Illustrating the transect algorithm. Left shows an irregular cloud of observed data points. Each open circle is a positive semi-definite $\Omega$ matrix on the Riemannian manifold. Right shows geodesics each with the margin of non-significance as curved lines. First transit path (major axis) in black (between the two points furthest distance from each other in the set), second transit path (next data major axis between new remaining furthest distance pair) in dark grey, third transit path (next remaining data major axis) in pale grey, and stippled remaining data points yet to be processed

**Fig. 36:**
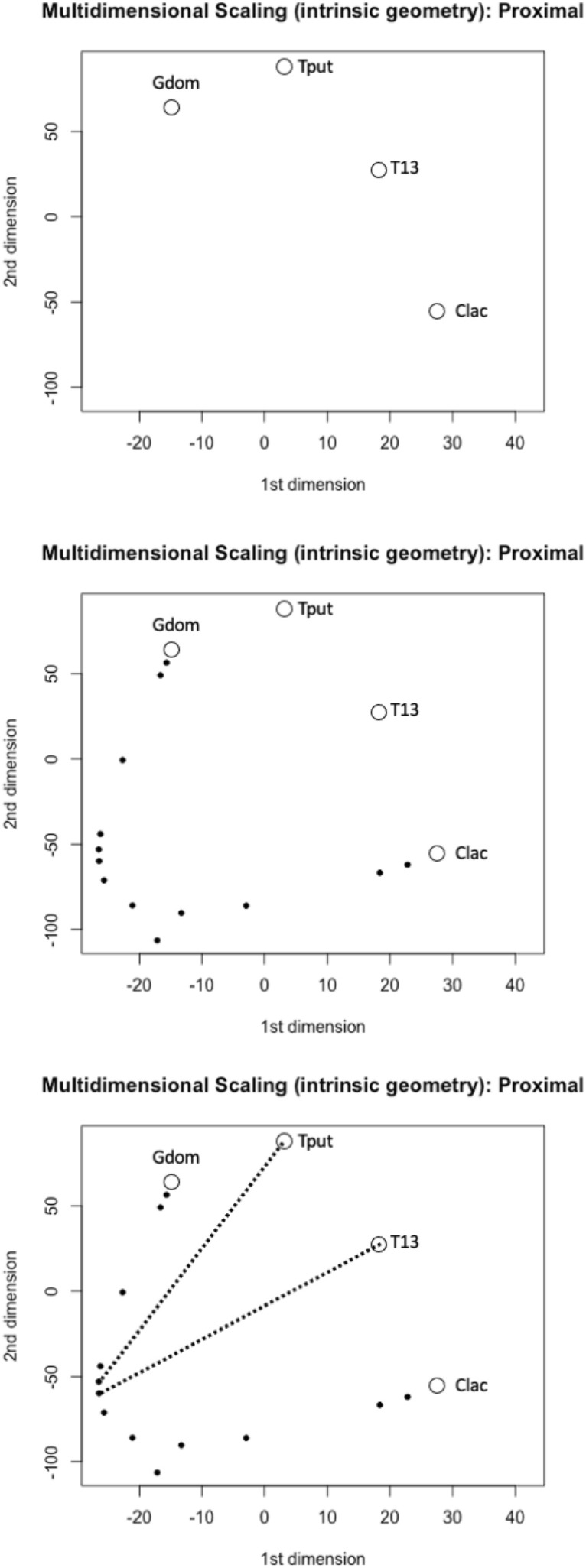
Ordination of Riemannian data from Bowman (2024b). Upper, Proximal to condyle moveable digit profile $\Omega$ matrices for chelae of reference extremal species *Glycyphagus domesticus* and *Carpoglyphus lactis* together with wild-collected and laboratory (T13) samples of *Tyrophagus putrescentiae* as open dots. Middle, interpolated matrices on geodesic (black dots). Lower, lines showing nearest interpolate to test samples. In both cases this line is significantly different than expected random variation. Lines drawn straight for simplicity of exposition - they themselves are curved geodesics

**Fig. 37:**
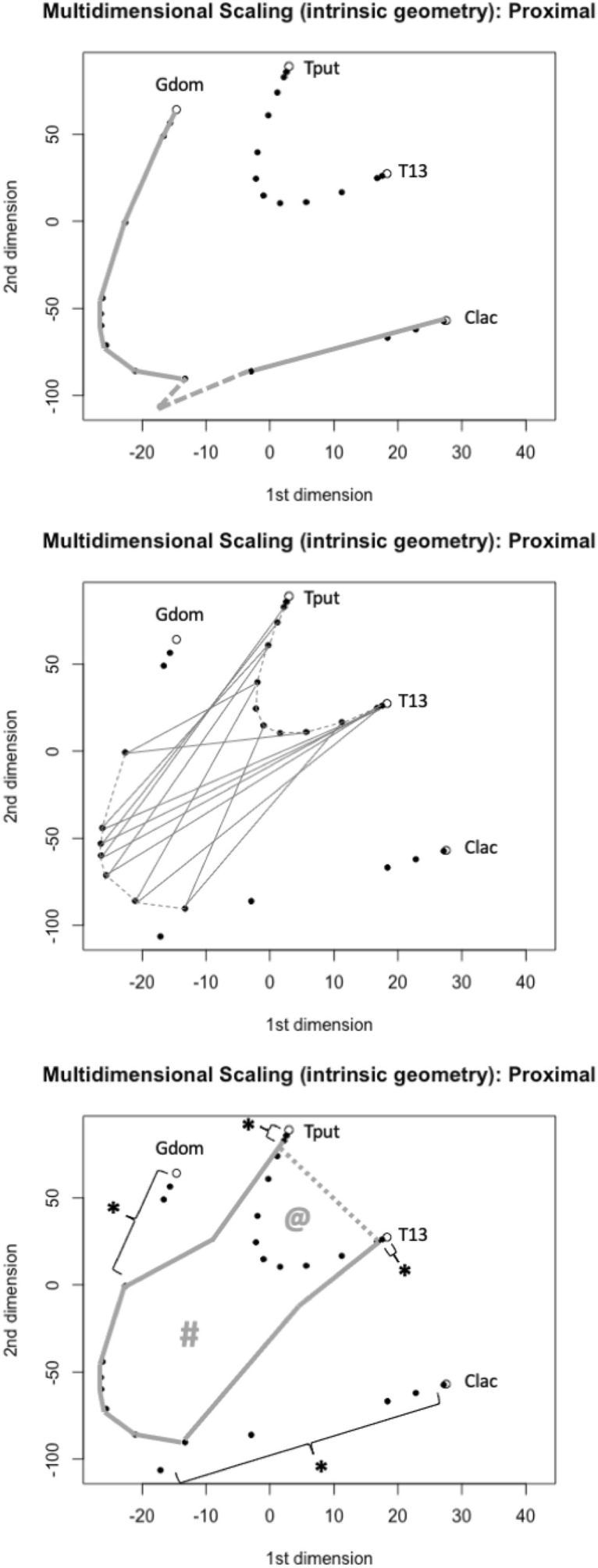
TRANSECT algorithm applied to data - see Fig. 36. Upper, STEP 1 & 2 completed, ’first candidate transit path’ in grey solid line (note calculation transient as chelal moveable digit profile swops from predominantly negative in *Carpoglyphus lactis* to essentially positive in *Glycyphagus domesticus*). Critical region set as $1.645\sigma _{proximal} = 6.474142$ from Bowman 2024b). STEP 3 calculate next the ’second candidate transit path’ as black dots in upper right of graph. Middle, STEP 4 data exhausted, STOP. CHECK if any $\Omega$ matrix effectively ’sits’ on more than one geodesic path. No original $\Omega$ matrices do, but solid lines indicate interpolated matrices that are not distinct between the two candidate paths (where illustrated as dashed lines). Lower, region of non-significance (#) as encompassing hull in thick grey line. Dashed line in grey indicates bound of extra region (@) of non-significance after final full checks. Straight lines used for simplicity of exposition - they all are curved geodesics. Two dimensional Euclidean embedding distorts true ’null’ region. However, each taxon has potential zones of distinct profile shape (indicated by asterisks)

**Fig. 38:**
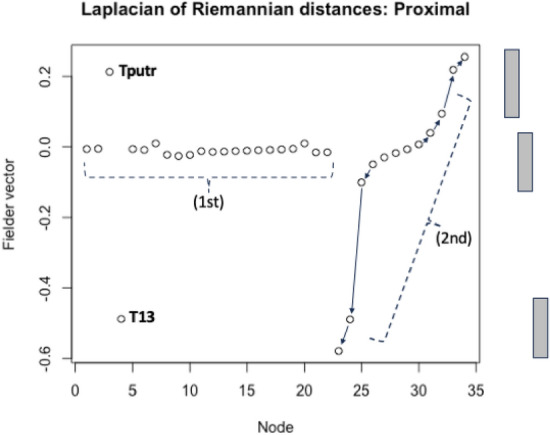
Eigen analysis of Laplacian from pilot example of moveable digit profile (proximal to condyle region - see Fig. 36). Node = notional number for each dot in Fig. 37. *y*-axis is coefficient of Fiedler vector (= vector matching second smallest non-zero eigenvalue of Laplacian). Grey bars indicate range of coefficients matching the three groups of $\Omega$ matrix nodes. Note chain of nodes on each candidate path. In particular the last few to either wild-collected *Tyrophagus putrescentiae* or laboratory sample T13 on the second geodesic

**Fig. 39:**
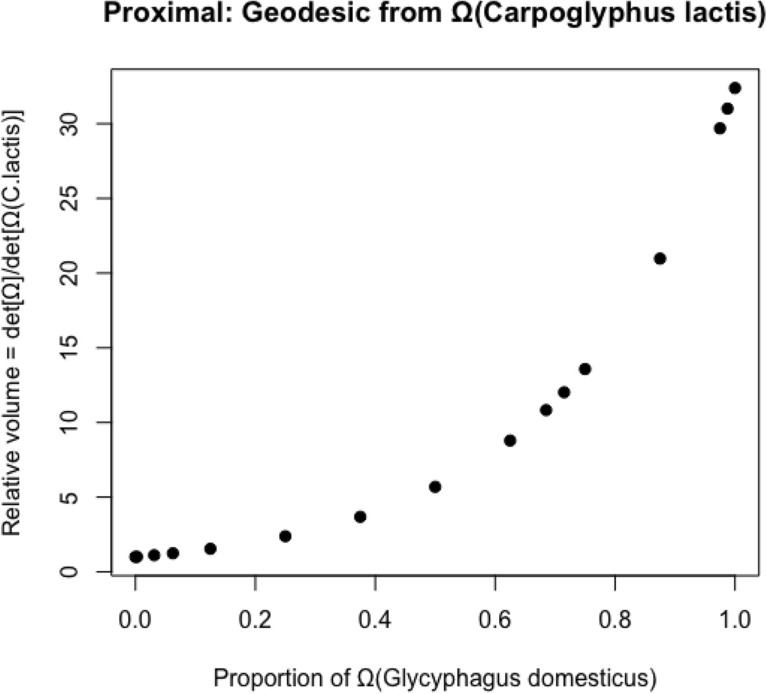
Relative volumes of $\Omega$ matrices along a geodesic. Data from Bowman (2024b) for moveable digit profile proximal to the articulating condyle (i.e., ascending ramus + basal ramus)

## Data Availability

Alcohol preserved samples of mite laboratory cultures have been deposited at: the British Museum (Natural History), London UK under accession number AQ ZOO 2020-78, and the Museum of Zoology, University of Michigan, Ann Arbor, MI USA under accession number ITR-UMMZ-I-2020-018. Collection, analysis and reporting of this data was self-funded. All new data generated or analysed, plus any model specifications are included in this published article, or in compliance with EPSRC's open access initiative are available via the Oxford Research Archive at 10.5287/ora-o5e1jdvjo.

## Appendix

**Table 1:** Free-living astigmatans reported as common in bird nests available as laboratory samples (from Hughes 1976, Solarz et al. 2004), Kew et al. (2015) grouped by genus

Family	Taxon	‘Presence in other habitats’	Population
Acaridae			
	*Acarus farris*	0100100111	A17
	*Acarus gracilis*	1000000000	A4
	*Acarus immobilis*	0000000101	A1 *
	*Aleuroglyphus ovatus*	0100100010	AL2 $\dag$
	*Rhizoglyphus robini*	0000000001	R1 $\dag$ *
	*Thyreophagus entomophagus*	0000000010	TH3 *
	*Tyrolichus casei*	0100000110	T62 $\dag$ *
	*Tyrophagus longior*	0000100101	T40 *
	*Tyrophagus palmarum*	0000000101	T17 *, T32
	*Tyrophagus putrescentiae*	0000010110	T13
	*Tyrophagus similis*	0000000101	T21 *, T44
Chortoglyphidae			
	*Chortoglyphus arcuatus*	0011100010	CH1 $\dag$
Glycyphagidae			
	*Glycometrus hugheseae* $\ddag$	0000000000	G3 $\dag$
	*Glycyphagus domesticus*	1011001110	G5 $\dag$
	*Lepidoglyphus destructor*	0100100011	G6 $\dag$ *
Pyroglyphidae			
	*Dermatophagoides pteronyssinus*	0011000000	D3
Suidasidae			
	*Suidasia pontifica* $\ddag \ddag$	0000000110	S5

$\ddag =$ was *Austroglycyphagus geniculatus*, $\ddag \ddag =$ was *Suidasia medanensis*. ‘Presence in other habitats’ records from Hughes (1976) not including feathers, nor fruit. Number string: 1 = positive, 0 = negative, in the order: Bat roosts, Mammal nests, Mattresses, Dust, Broiler houses, Cadavers (Braig and Perotti 2009), Meat, Cheese, Storage, Grassland. Population ex Bowman (2021). $\dag$ = Omnivore (others are Fragmentary feeders). * = Surface habit (others are Interstitial)

### Explanatory appendix

The analytical method is presented in terms of European Commission Horizon 2020 Technology Readiness Levels (https://ec.europa.eu/research/participants/data/ref/h2020/wp/2014_2015/annexes/h2020-wp1415-annex-g-trl_en.pdf). Bowman (2024b) already introduced that positive semi-definite matrices, such as those summarising the pattern of mite moveable digit profiles, exist not on an Euclidean surface but on a Riemannian manifold. However, another explanation plus ‘toy’ example can be found in Shabazi et al. (2021).

#### TRL1 concept

One wishes to partition the trends in digit profile patterns over the available ‘cloud of data’ (e.g., Fig. 35) into a cascade of distinct (two-way reversible) candidate paths transecting the observed multi-dimensional data space. This should be without constraining subsequent paths to necessarily pass through a reference point like the intrinsic mean of the data, nor such paths to be in any special way ‘independent in some sense’ to previous geodesic sub-manifolds like in Principal Geodesic Analysis (or its linearisations and probabilisitic formulations Fletcher et al. 2004, Fletcher and Joshi 2007, Zhang and Fletcher 2013, Sommer et al. 2014). Other methods like Principal Symmetric Space Analysis (Curry et al. 2019) could be used.

In two dimensions, the philosophy applied here is analogous to (but not the same as) that of fitting a linear regression line ($y=a.x+b$) defined by two chosen *extreme* points, and examining the points with ‘non-zero absolute residuals’ > ‘noise’ given that relationship, but then fitting a *further* relationship to explain *just* those ‘data points of disparity’, and repeating this transection to data exhaustion. It is thus then a partioning (or segmentation) algorithm

The justification for this particular approach is to consider that the envisaged acarine ‘data cloud’ arises from a *non-homogeneous* set of taxa of different phylogenies to which *different* ecomorphological relationships may separately apply at different taxonomic levels. The latter evolutionary relationships may not be represented in every taxon and some may be unique to a particular clade. The samples are thus not necessarily (stochastically varying) exchangeable units, yet they may still show a parsimonious set of common trends of interest to an evolutionary investigator.

In that way, this ‘toy’ disassemblative approach is a local ‘stripping’ algorithm (effectively ’backwards in time’) on the cascade of apomorphies from an ancestral plesiomorphic ground-state (near the $intrinsic\ mean(\Omega )$) for this habitat which is being built upon in different morphological directions ($\equiv$different segments) during the course of evolution. This is in contrast to decomposing data by a more normally expected global ‘fitting’ algorithm (like PGA, which is applicable only in the presence of one manifold with unknown structure) to describe the *universal* changes applied to all of its stated acarine inhabitants exchangeably over time. The output from the algorithm is thus a type of clustering process, where the local geometry of each candidate pathway could be different (as in the methods of Goh and Vidal 2008).

#### TRL2 pseudo-code

The ‘TRANSECT’ ALGORITHM is:

STEP 1. Calculate (over the whole set of *N* samples) the pair-wise geodesic distances

$$D_{i,j}=||\ log[\Omega _{i}^{-1/2} \Omega _{j} \Omega _{i}^{-1/2}]\ ||\ \ \ \ i \ne j$$

where $\Omega _{i}$ and $\Omega _{j}$ are the two sampled positive definite matrices under consideration (e.g., that for say the *i*th species sample and the *j*th species sample) and ||....|| means ‘norm of’.

Rationally set a margin for the maximum distance which is acceptable for an $\Omega$ matrix to be considered to effectively ‘fall’ on a specified geodesic (see Bowman 2024b). ‘Effectively falling’ means that the $\Omega$ matrix is ‘non-significantly’ different from the closest matrix interpolate on the line, Set $pathcounter = 0$

STEP 2. Find the ‘diameter’ of the data cloud (under consideration) i.e., $max(D_{i,j})$ over the set of *D*’s and then assign $m=i$ and $n=j$. Transit along the geodesic from these ‘eccentrics’ ($\Omega _{m}$ to $\Omega _{n}$) calculating the distance from the closest matrix interpolate to each of the remaining $(N-2)$ matrices $\Omega _{k}\ k\ne m, \ k\ne n$. Denote this geodesic as the (next) candidate ‘transit path’. Set $pathcounter = pathcounter+1$

STEP 3. Exclude from the current set (of *N*) $\Omega$ matrices: $\Omega _{m}$, $\Omega _{n}$ and any ‘non-significant’ $\Omega _{k}$, deeming them as ‘explained’ by that candidate ‘transit *pathcounter*’. Reset *N* to be *N* minus the number of $\Omega$ matrices removed from the set.

STEP 4.

- If there are no $\Omega _{k}$ matrices left (i.e., $N=0$, i.e., there is data exhaustion), STOP, concluding that all the matrices are ‘explained’ i.e., they fall on the accumulated set of transit paths somewhere.
- If there is one $\Omega _{k}$ matrix left (i.e, $N=1$), STOP, concluding that the matrices fall on the accumulated set of transit paths plus the (additional unique) transit path from the closest matrix interpolate on the last candidate transit path and $\Omega _{k}$.
- If there are two $\Omega _{k}$ matrices left (i.e, $N=2$), STOP, concluding that that the matrices fall on the accumulated set of transit paths plus the (additional unique) transit path between the last two matrices left.
- If there are $> 2$ $\Omega$ matrices left in the non-explained set, (i.e., $N> 2$) then just for that reduced number of ‘so far’ non-explained set of matrices GO TO STEP 2.

NOTE: A species not being on a particular geodesic path could be considered as being ‘significantly different’ to the evolving form of that candidate transit path. Clearly a large margin will mean all matrices are deemed to be explained by the first geodesic. A very small margin will decompose the $\Omega$ cloud into a maximum number of candidate paths. A very very small margin regards all the matrices as unmapped singletons. Comparison of the margin to the ‘radius’ of the data (i.e., $min(D_{i,j})$ over the full set of *D*’s) should be made (as the margin in a statistical sense is controlling the ‘Type 1 error’ of the procedure). Candidate transit paths are a type of regionalisation or clustering of connected subsets on the Riemannian manifold. Ties should be broken cognoscent of phylogeny, by matrix merger or randomly depending upon which is appropriate.

Before final conclusions are made, A CHECK should be made to see if any $\Omega$ matrix sits on more than one geodesic path (e.g., the two samples in centre of Fig. 35). These may be of even greater interest to evolutionary biologists than the *extrema* as the intersection may indicate common ‘unstable’ evolutionary areas (within dietary generalist functional groups). Or by examination of *post hoc* profile reconstructions and comparison to that of the $intrinsic\ mean(\Omega )$, these areas may represent original ‘ground morphological states’. Intersections here is only meant in the sense that they cross *within* the space of the ‘data cloud’ (as of course in a Riemannian space every pair of ‘straight-line’ geodesics must cross somewhere). Paths can be illustrated and explored using the methods deployed in Bowman (2024b).

The ‘taxon richness’ of paths could be assessed by the number of taxa included in each. Their taxon sparseness could be assessed by their diameter of the taxa within them compared to the radius of the taxa within them i.e., $\frac{max(D_{i,j,k})}{min(D_{i,j,k})}$ for the *k*th candidate path. Their taxon density could be assessed perhaps by the number of taxa included in each divided by their maximum geodesic length.

If the $\Omega$ matrices are variance scaled mean centred sums of squares and cross-products ‘correlation’ matrices then: comparisons as to their orthogonality to each other can be made with Herdin et al. (2005), and hypothesis tests versus specified alternatives made using Kullback–Leibler divergences (Tumminello et al. 2007). Allowance for the lack of independence due to shared ancestry in specific comparisons between taxa can be made by the use of a phylogenetic covariance matrix adjustment (under a Brownian or other random motion model of evolution, see Adams 2014).

#### TRL3 pilot example

The data from Bowman (2024b) is used (Fig. 36).

The results are shown in Fig. 37. They match the conclusions of Bowman (2024b).

Any taxon ordinating in the ‘null region’ has a ‘common denominator’ moveable digit profile form (proximal to the condyle) and could in theory diverge evolutionarily down either of the two bidirectional paths. Note the apparent differences in ‘velocity’ of relative morphological change along the geodesics (i.e., the $\frac{d[distance]}{d[mixing\ proportion]}$ distorted by the dimensional flattening).

Not only are the two *T.putrescentiae* samples distinct from the (*G.domesticus* $\leftrightarrow$ *C.lactis*) 1st candidate path, but so are the last few interpolated $\Omega$ matrices at both ends of the 2nd candidate path geodesic (between the two *T.putrescentiae* samples).

This conclusion can be checked by treating the pairwise Riemannian distances as representing a fully connected undirected graph. Edge weights in this graph are taken to be $\frac{1}{distance}$ between each of the $\Omega$ matrix nodes. The Fiedler vector (Fig. 38) of the Laplacian $L=D-A$ (where *D* is the degree matrix, in this example here *diag*[34], and *A* is the edge-weight adjacency matrix of the graph) gives the sparsest cut through this graph being thought of as a network. The Fiedler vector is the vector with the second smallest eigenvalue. This second smallest eigenvalue is the algebraic connectivity of the network (https://en.wikipedia.org/wiki/Algebraic_connectivity. The number of zero eigenvalues match the number of components in the graph. As the elements $D_{i,i}$ are of similar size, no normalisation is done.

This re-mapping approach confirms a single connected graph component (as expected for a digit profile), three groups, and two distinct samples of *T.putrescentiae*. Note how the last few $\Omega$ matrix interpolates on the second geodesic candidate path are close to each actual *T.putrescentiae* sample. TRL3 is achieved.

Each $\Omega$ matrix in itself can be explored further (see Technical appendix).

### Technical appendix

#### Exploring $\Omega$ matrices

The relative sizes of $\Omega$ matrices can be compared. Their volumes are given by the (absolute value) of their determinant. So for example, using the moveable digit profile data (i.e., nearest to the condyle) from Bowman (2024b), the relative sizes of each interpolated $\Omega$ matrix along the geodesic from the consensus $\Omega _{Clactis}$ (left hand side) to the consensus $\Omega _{Gdomesticus}$ (right hand side) can be plotted (Fig. 39). One can see as one moves away from a more plesiomorphic form of the carpoglyphid (nearer the zero point of the multidimensional Riemannian ‘cone’ of positive semi-definite matrices) towards the more differentiated glycyphagid form, the volume monotonically increases.

Bowman (2024b) outlines decomposing the Riemannian metric ($log[\Omega _{a}^{-\frac{1}{2}}.\Omega _{b}.\Omega _{a}^{-\frac{1}{2}}])$ between two positive semi-definite matrices $\Omega _{a}$ and $\Omega _{b}$ by examining the rescaled relative eigenvectors of its matrix kernel ($\Omega _{a}^{-\frac{1}{2}}.\Omega _{b}\Omega _{a}^{-\frac{1}{2}}$) e.g., first component = $eigenvector_{1}.\sqrt{log(eigenvalue_{1})}$. This allows looking at directions along the geodesic from the view point of $\Omega _{a}$ or from the view point of $\Omega _{b}$ simply by examining the largest positive and largest negative *log*(*eigenvalues*).

Further specific (alternative hypothesis) profile patterns e.g., *y*[0] (and thus $\Omega _{a}=\Omega _{0}=\bar{y}[0].\bar{y}[0]$), can be deployed and the Riemannian distance (and directions) to or from say a data-derived $\Omega _{b}$, compared to the expected random variation in distance.

Now what if $\Omega _{a}=I$ (i.e., a $p\ by\ p$ identity matrix)?

Then $I^{-\frac{1}{2}}=I$ and so $\Omega _{a}^{-\frac{1}{2}}.\Omega _{b}.\Omega _{a}^{-\frac{1}{2}} \Rightarrow \Omega _{b}$. This means that the (first component of the) direction along a (new) geodesic towards $\Omega _{b}$ away from the identity matrix *I* can be given by the $\Omega _{b}$ matrix (relative) $eigenvector.\sqrt{log(eigenvalue)}$ matching the largest positive value for *log*(*eigenvalue*). Accordingly, the direction along that geodesic but now away from $\Omega _{b}$ towards the identity matrix *I* can be given by the original $\Omega _{b}$ (relative) $eigenvector.\sqrt{log(eigenvalue)}$ with the largest negative value for *log*(*eigenvalue*). The same argument applies for any $\Omega _{a}$ when $\Omega _{b}=I$. Note that the eigenvectors do not fundamentally change simply their scale. This of course is consilient with the widespread practice of considering the largest eigenvalues from $SVD(positive\ semi\text {-}definite\ matrices)$ as of interest.

This now allows a way to further examine the individual $\Omega$ matrices. However, in the context of moveable digit profiles what does $\Omega =I$ mean?

Bowman (2024b) explains that $\Omega$ comprises a term for the macro-scale mean profile (i.e., $\bar{y}.\bar{y}^{T}$, where $\bar{y}$ are the projected profile means and $^{T}$ means transpose) plus a term for the stochastic micro-scale variation around that mean profile ($\Gamma$). If one assumes no such (co)variation (which then matches the zero off-diagonal elements in *I*), then assuming $\Omega =I$ simply means a fixed plesiomorphic moveable digit of uniform height (here $=1$ unit, but its overall scale can be lost into the overall magnitude of the set of eigenvalues).

In quantitative morphology, integration is when otherwise distinct parts of an organism function and/or evolve as one unit. Modularity is when an organism displays distinct regions (modules) within which integration is high and between which integration is low (Klingenberg 2014). So, any $\Gamma$ with very small absolute off-diagonal elements (i.e., very little covariation or correlation) infers those matching row and column projected points are logically at great ‘distance’ to each other in the stochastic growth processes behind them, which effectively sets their matching edge-weights to *zero* in a graph re-mapping for that projected location on the moveable digit profile, and therefore as a graph indicates disconnections. This will divide up the digit profile into possible distinct sections of ‘morphological integration’. Where these semi-independent regions are located may be of interest to evolutionary morphologists who may be investigating mosaicism (Felice and Goswami 2017) in the adaptive radiation of acarines.
